# Measures of identity in adolescents/young adults with long-term physical health conditions: a systematic review

**DOI:** 10.1093/jpepsy/jsag001

**Published:** 2026-02-13

**Authors:** Tessa Rugg, Line Caes, Christopher Eccleston, Bernie Carter, Jeremy Gauntlett-Gilbert, Clare E Pain, Abbie Jordan

**Affiliations:** Department of Psychology, University of Bath, Bath, United Kingdom; Centre for Pain Research, University of Bath, Bath, United Kingdom; Division of Psychology, Faculty of Natural Science, University of Stirling, Stirling, United Kingdom; Centre for Pain Research, University of Bath, Bath, United Kingdom; Faculty of Health, Social Care and Medicine, Edge Hill University, Ormskirk, United Kingdom; Bath Centre for Pain Services, Royal United Hospital, Bath, United Kingdom; Centre for Health and Clinical Research, University of the West of England, Bristol, United Kingdom; Department of Paediatric Rheumatology, Alder Hey Children’s NHS Foundation Trust, Liverpool, United Kingdom; Institute of Life Course and Medical Sciences, University of Liverpool, Liverpool, United Kingdom; Department of Psychology, University of Bath, Bath, United Kingdom; Centre for Pain Research, University of Bath, Bath, United Kingdom

**Keywords:** identity, young adults, long-term physical health conditions, measure, COSMIN

## Abstract

**Objective:**

We identified and evaluated measures of identity used with adolescents/young adults aged 16–24 years living with long-term physical health conditions (LTC-P), focusing on the conceptualization, development, and psychometric properties of measures. Review funded by Sir Halley Stewart Trust and the University of Bath. Review protocol: https://osf.io/bhkze.

**Method:**

Five databases (APA PsychNET, PubMed, Web of Science, SCOPUS, and CINAHL) were searched. Studies were included if they were peer-reviewed, reported participants aged 16–24 years with LTC-P, and used a quantitative identity measure. Psychometric properties and risk of bias were evaluated using Consensus-Based Standards for the Selection of Health Measurement Instruments V1 (COSMIN) quality criteria, and content validity was reviewed narratively. Data analysis and synthesis followed COSMIN methodology for reviews.

**Results:**

Thirty-seven papers met inclusion criteria, involving 9,486 participants and 16 identity measures. Across the papers, identity was defined and conceptualized in varied ways. Only three measures, the Illness Identity Questionnaire (IIQ), Dimensions of Identity Development Scale (DIDS), and Identity Motives Scale (IMS), were used in multiple studies and assessed for psychometric quality. Evaluated properties included structural validity, internal consistency, measurement invariance, and construct validity. The IIQ and DIDS were tentatively recommended for use. The IMS was rated as needing further validation due to limited content validity.

**Conclusions:**

The IIQ and DIDS may be appropriate when their conceptual focus aligns with research objectives. Remaining measures should be used cautiously; many lack developmental or condition-specific relevance. Future identity measure development should integrate lived experience, expert input, and rigorous psychometric testing to ensure tools are both meaningful and fit for purpose in target populations.

Long-term physical health conditions (LTC-P) are defined as lasting for 3 months or longer for which symptoms persist, often resulting in the limitation of normal activity, and requiring access to specialized health resources and require self-management (Moore et al., 2019). Although adolescence/young adulthood is typically perceived as a period of good physical health, and LTC-P prevalence often increases with age (Watson et al., 2025), a significant number of adolescents/young adults (AYA) live with LTC-P. Data from England (NHS England, 2024) suggest that 45% of 16–24-year-olds have a long-term physical or mental health condition, disability, or illness. With regard to defining AYA, definitions of the age range differ according to context or discipline. Our chosen perspective encapsulates late adolescence (Sawyer et al., 2018) and emerging adulthood (Arnett, 2000), encompassing ages 16–24 years. It is around this age at which AYA become more autonomous (Pfeifer & Berkman, 2018), and those with LTC-P may start to take more responsibility for their care and transition into adult services (Coyne et al., 2019).

There is a growing body of literature examining the effects of living with LTC-P on AYA’s personal and social development, with an increasing focus on personal identity. Personal identity refers to an individual’s sense of self-consistency and coherence across time and life contexts (Branje et al., 2021) and comprises multiple content domains (e.g., relationships, hobbies, education) that shape one’s overall sense of self (Galliher et al., 2017). Although identity is subjective, it is also shaped by social interactions and environments. Relationships with peers (Pugh & Hart, 1999; Sugimura et al., 2022) and family (Albert Sznitman et al., 2019; Crocetti, 2017) are instrumental in shaping identity development. For AYA negotiating a sense of self, social connectedness can provide reassurance, strengthen confidence, and support the development of a stronger sense of identity (Ja & Jose, 2017; Noble-Carr et al., 2014). However, AYA living with LTC-P often face substantial barriers to accessing these crucial opportunities for social connection. Physical limitations and/or symptom management requirements can hinder active participation in peer activities (Pinquart & Pfeiffer, 2015), potentially leading to social isolation and stigma that contribute to feelings of marginalization and emotional distress (Qualter et al., 2021; Spencer et al., 2023). Educational environments also contribute positively to identity formation, through deliberate educational practices and guidance from teachers, and/or informal interactions with peers (Lannegrand-Willems & Bosma, 2006; Verhoeven et al., 2019). However, accessing these opportunities for identity formation may be especially challenging for AYA with LTC-P who may have significant absences or reduced attendance (Alsaggaf & Coyne, 2020), further complicating their identity development process.

One of the most significant developmental changes experienced by individuals with LTC-P is the loss of self, as they observe their former identities fade, often without the emergence of new, equally meaningful identities (Charmaz, 1983). For example, AYA living with chronic pain often perceive themselves as lagging behind their peers in identity development, as well as experiencing identity disruption (Eccleston et al., 2008; Jordan et al., 2018). For those who have lived with LTC-P since early childhood, identity challenges may involve negotiating a sense of self in comparison to healthy peers and managing the ongoing demands associated with their condition (Commissariat et al., 2016).

Many studies have shown the varying ways in which LTC-P can become integrated into AYA’s self-concept (Di Risio et al., 2011; Jordan et al., 2018; Vanderhaegen et al., 2024), as well as apprehensions about how their condition may influence their future identity (Jones et al., 2020).

Identity in the context of LTC-P has been explored through theoretical frameworks such as illness identity (Oris et al., 2016) and the dynamic model of identity reconstruction (Yoshida, 1993). Illness Identity Theory (Oris et al., 2016) describes how individuals integrate a chronic illness into their sense of self. Studies have shown that *acceptance* (illness is recognized and integrated into identity without dominating it) and *enrichment* (illness is seen as an opportunity for personal growth) are associated with better treatment adherence (Commissariat et al., 2020; Rassart et al., 2021), while *engulfment* (illness dominates identity) is related to more depression, lower life satisfaction, and poorer health (Rassart et al., 2021, 2023). While this theory was initially developed with AYA, the model itself is not age-specific and has been applied to adult populations. Yoshida’s (1993) dynamic model of identity reconstruction conceptualizes the adjustment to LTC-P as a pendulum-like process, in which individuals swing between their pre-illness identity and the realities of their post-illness self. Yoshida proposes that identity reconstruction is categorized into five categories, namely: (1) *the former self*, (2) *the supernormal identity*, (3) *the disabled identity as total self*, (4) *the disabled identity as an aspect of the total self*, and (5) *the middle self*. Although developed from adult experiences of chronic illness (specifically spinal cord injury), this framework has been expanded by Stepleman et al. (2017), who identified that higher scores on *reactionary identity* (in which individuals try to prove self is the same, or even better than before illness, and illness dominates self-perception) were associated with greater perceived stigma and higher levels of depression in an adult multiple sclerosis (MS) population. Together, these frameworks highlight the ways in which living with LTC-P can shape identity over time and provide valuable conceptual foundations for developing measures that can assess key identity processes. Robust measures can allow researchers to monitor identity development across different populations and contexts, as well as assist clinicians in identifying and helping with identity-related challenges.

Although a sizable number of measures are available to examine identity development, there has not yet been a comprehensive evaluation of their psychometric properties and applicability in the context of AYA living with LTC-P. Furthermore, there is also a lack of understanding regarding which LTC-P populations are represented in existing quantitative research. Such insights can inform future research directions and facilitate more targeted and effective identity assessment among AYA living with LTC-P.

Therefore, our aim with this systematic review was to identify existing measures of identity in AYA with LTC-P, and to what degree they have been psychometrically evaluated, as defined by the Consensus-Based Standards for the Selection of Health Measurement Instruments V1 (COSMIN; Mokkink et al., 2018). The review was guided by the following research questions:

1. In AYA aged 16–24 with LTC-P, what measures have been used to assess identity?
2. What are the psychometric qualities of the existing identity measures?
3. How were the existing identity measures developed?
4. What are the limitations of the existing identity measures?

## Methods

### Protocol and registration

The study protocol is available through the Open Science Framework, and there were no deviations from the protocol: https://osf.io/y856n. This review is reported using recommendations from Preferred Reporting Items for Systematic Reviews and Meta-Analysis (PRISMA; Page et al., 2021), and the review of measures follows an adapted version of COSMIN (Mokkink et al., 2018).

### Eligibility criteria

This review specifically focused on measures that examined personal identity, defined as an individual’s sense of self, rather than other forms of identity such as social, gender, ethnic, or professional identity.

To be included for review, papers were required to have been empirical and to have adopted quantitative or mixed methods. Papers were required to have been (1) published in peer-reviewed journals, (2) written in the English language, (3) include at least one measure of identity formation, and (4) include participants aged 16–24 years with a diagnosed or self-diagnosed LTC-P. Papers were included if they had participants in this age range, regardless of whether or not the results were reported separately for the 16–24-year-old age group.

Papers were excluded from the review if they did not collect primary data, included populations in which the primary focus was on (1) mental health conditions (e.g., depression, anxiety, obsessive compulsive disorder, etc.), (2) neurodevelopmental conditions (e.g., autism, attention-deficit hyperactivity disorder, Tourette syndrome), or (3) cognitive impairment (e.g., Down’s syndrome, traumatic brain injury).

### Information sources

Searches were conducted on October 24, 2024, in the following databases: APA PsychNET, PubMed, Web of Science, SCOPUS, and CINAHL. No restrictions were put on the search strategy in terms of date. Additionally, forward and backward citation searches for all eligible papers were conducted using Google Scholar.

### Search strategy, paper selection, and data extraction

With guidance from a specialist subject librarian, a search strategy (Table 1) was devised that aimed to capture three main concepts: (a) personal identity, (b) LTC-P, and (c) measures of personal identity development. To fully capture the recent expansion of the age boundary of adolescence (Sawyer et al., 2018), no age-related concept was included in the search terms. Restricting the search to words associated with AYA may have excluded papers that conceptualize this group as 18 years and below. Instead, this inclusion criterion was screened in either the Title/Abstract stage, or the full-text review. The main concepts of personal identity, LTC-P, and measures of personal identity development were limited to searching in the Title/Abstract fields only. The full-search strategy for each database can be found in the online supplementary material 1.

**Table 1.** Search terms.

**Identity:** Identity OR “Identity Formation” OR “Identity Development” OR “Identity Exploration” OR “Illness Identity” AND
**Long term conditions—physical:** “Long Term Condition” OR “Chronic condition” OR LTC OR “Recurrent Condition” OR “Chronic Disease” OR “Chronic Pain” OR “Persistent Pain” OR Diabetes OR Crohn’s OR IBD or “Ulcerative Colitis” OR “Inflammatory Bowel Disease” OR CRPS OR “Complex Regional Pain Syndrome” OR Fibromyalgia OR Arthritis OR Endometriosis OR Migraine OR “Chronic daily headache” OR Osteoarthritis OR “Spina Bifida” OR Asthma OR Allergy OR “Atrial fibrillation” OR “Chronic Constipation” OR “Chronic Fatigue Syndrome” OR CFS OR ME OR “Myalgic encephalomyelitis” OR “Chronic Kidney Disease” OR “Congestive Heart Failure” OR Epilepsy OR Hypertension OR HIV OR “Irritable Bowel Disease” OR “Multiple Sclerosis” OR Anaemia OR Angiooedema OR “Sickle Cell” OR “Autoimmune Disorder*” OR “Autoimmune Disease” OR Lupus OR “Sjögrens syndrome” OR “Bronchopulmonary dysplasia” OR Cancer OR “Cardiac arrhythmias” OR “Coeliac Disease” OR “Coronary heart disease” OR “Cystic Fibrosis” OR “Endocrine disorder*” OR “Widespread pain” OR Gout OR “Heart Failure” OR “Lung Fibrosis” OR “Ehlers Danlos syndrome” OR haemophilia OR Rheumatoid OR Neurofibromatosis OR “Charcot-Marie-Tooth disease” OR Scoliosis OR scleroderma OR vasculitis OR “juvenile idiopathic arthritis” OR “rheumatoid arthritis” OR “congenital heart disease” OR thalassemia OR “chronic liver disease” OR cancer OR dermatitis OR eczema OR psoriasis AND
**Measure:** Surveys OR Questionnaires OR Psychometrics OR measure* OR tool* OR assessment OR scale* OR inventory OR index

Papers retrieved from searches were uploaded into Covidence (2024), an online systematic review tool, and duplicate papers were removed. In the first instance, all papers were screened by the first author (T.R.), with a second reviewer (H.J.) independently screening at least 50%. Any disagreements were resolved by a third reviewer (A.J.). Any papers for which eligibility was not clear from the title/abstract alone were included at this stage. After this initial screening process, included papers were assessed for eligibility via retrieving and reviewing the full text. Full-text papers were independently reviewed by two reviewers (drawn from T.R., A.J., and H.J.), with disagreements again being resolved by a third reviewer (L.C.).

Data extraction was then performed independently by two reviewers of the study team (drawn from T.R., L.C., and F.T.), using a piloted data extraction form that was hosted within Covidence (2024). Data extracted included information regarding: the paper (date, authors, journal name, country of origin), study information (definition of identity used, participant age range, gender identity, sex, sexual orientation, ethnicity, race, socioeconomic status (SES), religion, LTC-P, study design, recruitment site), measure information (name, purpose, target population, translations, manual included, theories/concepts captured), and any COSMIN psychometric evaluation outcome properties available (PROM development, content validity, structural validity, internal consistency, cross-cultural validity/measurement invariance, reliability, measurement error, criterion validity, hypotheses testing for construct validity, and responsiveness). When data were missing or unclear, the first author of the paper was contacted, with subsequent authors approached if no response was obtained after two attempts.

### Study quality and risk of bias

A two-step approach to assess the quality of each identity measure was conducted. First, all measures were rated using the Cohen et al. (2008) criteria for evaluating evidence-based assessment, identifying measures as either “well-established,” “approaching well-established,” or “promising assessment.” To achieve a rating of well-established, measures must have been presented in at least two peer-reviewed papers by different researchers or research teams, with sufficient detail available about the measure to allow for critical evaluation or replication (e.g., a manual provided or available upon request) and detailed statistics indicating good validity and reliability in at least one peer-reviewed paper. To achieve a rating of approaching well-established assessment, measures must have been presented in at least two peer-reviewed paper by different or the same researcher or research team, with sufficient detail available about the measure to allow for critical evaluation or replication (e.g., a manual provided or available upon request) with validity and reliability information presented in either vague terms (e.g., no statistics presented) or moderate values. Finally, measures rated as promising assessment were presented in at least one peer-reviewed article, with sufficient detail to allow critical evaluation and replication (e.g., a manual provided or available upon request), and reliability information presented in either vague terms (e.g., no statistics presented) or moderate values.

In Step 2, measures rated “well-established” and “approaching well-established” using the Cohen criteria were assessed for quality using an adapted COSMIN checklist version 1.0 (Mokkink et al., 2018). Data were independently extracted from each paper containing each measure by three reviewers (T.R., F.T., and L.C.). These data were then rated independently by two reviewers (T.R. and L.C.) on structural validity, internal consistency, cross-cultural validity, measurement invariance, reliability, construct validity, and criterion validity using the COSMIN Risk of Bias checklist, v1.0.

In the first step, the quality of the methodological approach of each paper in assessing a particular COSMIN measurement property was rated using a four-point rating system, ranging from “very good,” “adequate,” “doubtful,” or “inadequate.” The overall quality rating of the paper was then determined by taking the lowest rating of any COSMIN measurement property the paper addressed, with this rating used to grade the quality of the evidence for each measure (e.g., a measure with “inadequate” evidence will decrease trust in the overall conclusions of the measurement property). If information regarding a particular measurement property in the checklist was missing, then the rating was left blank.

In the second step, each paper’s evidence for a given measurement property was rated as sufficient (+), insufficient (–), or indeterminate (?) (see online supplementary material 2 for criteria). Any disagreements between the two reviewers were discussed, and a consensus was made. Next, to determine an overall rating for each measurement property of a measure, we summarized the ratings from all relevant papers. Following COSMIN guidance, a summarized rating was considered sufficient, insufficient, or indeterminate if at least 75% of the individual study ratings met that criterion. In the case of inconsistent ratings, overall ratings were based on the majority rating. In the case of an equal split between ratings, the decision was made on the ratings of the most recent publications assessing that measurement property of the measure.

In the final step, a modified Grading of Recommendations Assessment, Development, and Evaluation (GRADE; Mokkink et al., 2018) approach was used to downgrade evidence if there were any concerns about the quality of evidence presented, with evidence starting at high quality, and subsequently downgraded by one or two levels per factor. The factors comprised risk of bias (as assessed in the first step described above), inconsistency (of results across studies), indirectness (evidence coming from different populations than those of interest in current review), and imprecision (total sample size of studies included in review).

While the COSMIN checklist was used to assess other measurement properties, the criteria for evaluating content validity were found to be overly rigid for the purposes of this review, which has been noted previously (Rothmund et al., 2023; Smith et al., 2021). Applying COSMIN standards would have led to “inadequate” ratings for many measures, which we believe would not accurately reflect the efforts undertaken by the paper authors and lead to the dismissal of potentially valuable measures being used in the future. Instead, we chose to summarize how content validity was addressed in each paper in a narrative review, looking at item generation, content validity, and face validity, allowing readers to make their own informed judgments on potential use in future research of measures. This was performed by one reviewer (T.R.).

In the final step, each measure was categorized using an adapted COSMIN guideline: Category A (at least low-quality evidence for “sufficient” internal consistency), Category B (not meeting criteria for A or C), or Category C (high-quality evidence for an “insufficient” property). Category A measures are considered suitable for recommendation, Category B require further research, and Category C are not recommended. As a full COSMIN content validity assessment was not performed, Categories A and B should be interpreted with caution, and further research on content validity, including concept elicitation and stakeholder involvement, is advised.

A meta-analysis was not undertaken, as the aim of this review was to evaluate the development and psychometric quality of identity measures, rather than to synthesize outcome effects.

## Results

### Included studies

**Figure 1.**
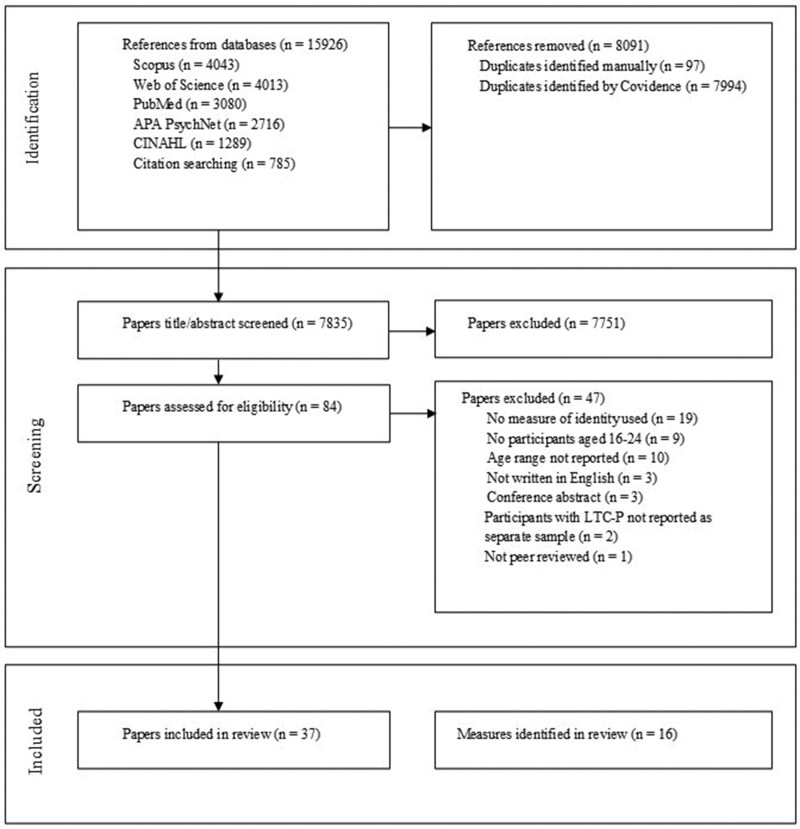
PRISMA flow chart. PRISMA = Preferred Reporting Items for Systematic Reviews and Meta-Analysis.

The electronic searches returned 15,926 records, with 8,091 duplicates removed. Of the 7,835 papers that remained, 7,751 papers did not meet the inclusion criteria after title/abstract screening, leaving 84 for full-text eligibility assessment. After full-text screening, 47 papers were excluded. The main reasons for exclusion included: (1) papers did not contain a measure of identity development (*n* = 19), (2) the age range of participants was not reported (*n* = 10), or (3) papers did not contain participants aged 16–24 years (*n* = 9). Figure 1 contains the study PRISMA flow chart. After screening, 37 papers measured identity and were included in the review. Within these papers, 16 unique measures were identified.

Cohen’s Kappa for the title/abstract screening was 0.51, signifying moderate agreement between reviewers. Most discrepancies arose from papers employing the Illness Perception Questionnaire (Weinman et al., 1996), of which the “Identity” subscale does not correspond to the construct of personal identity specified for this review. After discussion, the team agreed to exclude these papers from further analysis. Cohen’s Kappa for full-text screening was 0.84, indicating strong agreement.

### Overview of paper characteristics

Each paper was assigned a unique code, which is presented in Table 2 alongside a summary of the included papers. Papers were published between 1987 and 2024, with the majority after 2014 (*n* = 31). There were 16 LTC-P observed in the papers. Type 1 diabetes was the most investigated LTC-P (*n* = 10), followed by congenital heart disease (*n* = 9), HIV (*n* = 5), and MS (*n* = 5).

**Table 2.** Overview of studies included for review.

							Study population					
Code	Author (year), Country	Study design	Definition of identity given?	Recruitment site	Identity measure	Properties evaluated in study	Condition/s observed	Total n	**Age range (Mean, *SD*)/16–24 age range, *n*** ^a^	Sex/gender %	Sexual orientation %	Ethnicity/race %
01	Allebone et al. (2015), Australia	Cross-sectional	No	Patient pool of Comprehensive Epilepsy Program (CEP) at Austin Health in Melbourne, Australia.	The Ego Identity Process Questionnaire (EIPQ)	Internal consistency	Mesial temporal (MT) and non-mesial temporal (NMT) lobe epilepsy	51 (MT 19, NMT 32)	MT: 20–67 (36.63, 13.44)^a^ NMTL 23–63 (37.94, 10.64)^a^	Gender: MT Male 53; NMT Male 37.5	Not reported	Not reported
02	Andonian et al. (2021), Germany	Cross-sectional	No	German Heart Centre Munich	Illness Identity Questionnaire (IIQ)	Internal consistency, discriminant validity.	Congenital Heart Disease (CHD)	229	18–73 (38.2, 12.5)^a^	Gender: Male 55; Female 45	Not reported	Not reported
03	Andonian et al. (2020), Germany	Cross-sectional	No	German Heart Centre Munich	IIQ—German translation	Reliability, Structural validity, internal consistency	CHD	229	18–73 (38.2, 12.5)^a^	Gender: Male 55; Female 45	Not reported	Not reported
04	April et al. (2022), United States	Cross-sectional	Yes	Online support groups on Facebook and Reddit, and social media.	Identity change: amount and affective change	None	Lupus 10.7% (*n* = 37); rheumatoid arthritis 8.4% (*n* = 29); psoriasis 7.5% (*n* = 26); chronic fatigue syndrome 7.0% (*n* = 24), and other (e.g., psoriatic arthritis, fibromyalgia, hashimoto’s, Crohn’s disease, celiac disease, Lyme disease, mixed connective tissue disease, endometriosis) 66.4% (*n* = 229).	345	19–73 (34.08, 11.23)^a^	Gender: Female 60	Not reported	Race: White 77.8; mixed racial background 15.1; other 7.2
05	Berg et al. (2018), United States	Cross-sectional	No	Participants under the care of a pediatric cardiologist at University of Utah	The Dimensions of Identity Development Scale (DIDS)	None	Long QT syndrome, hypertrophic cardiomyopathy, dilated cardiomyopathy	12	12–18 (14.7, 2.06)^a^	Gender: Male 66.7; Female 33.3	Not reported	Not reported
06	Brener et al. (2020), Australia	Cross-sectional	No	Social media and email lists of Australian Federation of AIDS Organisations (AFAO), and the National Association of People Living with HIV Australia (NAPWHA).	HIV Centrality	None	HIV	181	21–83 (49.94, 11.41)^a^	Gender: Male 88; Female 11; Transgender 1	Gay/homosexual 71; Bisexual 6; Straight/heterosexual 17; Queer 3; Other 3	Not reported
07	Calandri et al. (2019), Italy	Cross-sectional	Yes	Multiple Sclerosis Clinic Centre (Regional Referral Multiple Sclerosis Centre (CRESM), Torino, Italy),	Identity Motives Scale	Internal consistency	Multiple sclerosis (MS)	66	18–30 (25.2, 3.4)^a^	Gender: Women 63.6	Not reported	Not reported
08	Calandri et al. (2018), Italy	Cross-sectional	Yes	Referral Multiple Sclerosis Centre (CRESM), Torino, Italy	Identity Motives Scale	Internal consistency	Multiple sclerosis (MS)	90	20–65 (37, 12)^a^	Gender: Women 55	Not reported	Not reported
09	Calandri et al. (2020), Italy	Cross-sectional	Yes	Referral Multiple Sclerosis Centre (CRESM), Torino, Italy	Identity Motives Scale	Internal consistency	MS	101	18–35 (26.9, 4.2)^a^	Gender: Men 31; Women 69	Not reported	Not reported
10	Carroll et al. (2020), United Kingdom	Cross-sectional	No	Online support forums and charity websites	IIQ	Internal consistency	Mayer–Rokitansky–Kuster–Hauser syndrome (MRKH)	263	16.1–74.4 (31.6, 11.22)^a^	Sex: Female 100	Heterosexual 79.4; LGBTQI+ 20.6	Ethnicity: White 84.5; BAME 16.4
11	Commissariat et al. (2023), United States	Cross-sectional	Yes	Pediatric diabetes centres (Joslin Diabetes Centre, Yale School of Medicine)	Accepting Diabetes and Personal Treatment (ADAPT)	Internal consistency, criterion validity and construct validity.	Type 1 diabetes (T1D)	165	13.2–25.8 (18.5, 3.2)^a^	Sex: Male 46.1	Not reported	Race/ethnicity: Caucasian 87.3
12	Earnshaw et al., (2015), United States	Cross-sectional	No	Drop in centers, housing programs, and other organizations providing services to people living with HIV	A modified version of the identity subscale of the Collective Self-Esteem Scale	Internal consistency	HIV	93	21–68 (50.28, 8.55)^a^	Gender: Male 59.1; Female 37.6; Transgender 3.2	Heterosexual 81.7; Gay, lesbian and/or bisexual 15.1	Ethnicity: Latino(a) 38.7 Race: Black 55.9 l White 19.4; Other 18.3
13	Gavaghan & Roach (1987), United States	Cross-sectional	Yes	Hospital clinics (cancer patients), University and high schools (healthy control)	Ego Identity Incomplete Sentence Blank (EI-ISB)	Interrater reliability, Construct validity, Criterion validity	Cancer—leukemias, lymphomas, sacromas, Wilm’s and other tumours.	42	14–22 (17.5)^a^	Male *n* = 19; Female *n* = 23	Not reported	White 93; Black 7
14	Geuens et al. (2023), Belgium	Cross-sectional	Yes	Participants were selected from the database of the Neuromuscular Reference Center for Children (NMRC) of the University Hospitals Leuven	IIQ	Internal consistency	Neuromuscular disorders (NMD) and Type 1 diabetes mellitus (DM)	NMD 59; T1D 118	NMD 12–22 (15.92, 2.90); T1D 14–22 (16.31, 2.41^a^	NMD Men 69.5; Women 30.5 T1D Men 69.5; Women 30.5	Not reported	Not reported
15	Golub, Rendina & Gamarel (2013), United States	Cross-sectional	No	Gay, lesbian, and bisexual (GLB) community events in New York City (NYC) in the fall of 2006 and the spring of 2007	Impact on Self Concept Scale (ISCS)	Construct validity, internal consistency	HIV	129	18–54 (42.46, 10.12)^a^	Sex: Men 100	Not reported	Race/ethnicity: White 47; Black 23; Latino 25; other race/ethnicity or multiple races 5
16	Graziano et al. (2020), Italy	Cross-sectional	Yes	Regional Referral Multiple Sclerosis Centre (CRESM), Torino, Italy	Identity Motives Scale	Internal consistency	MS	74	19–57 (37.7, 10.7)^a^	Sex: Women 100	Not reported	Not reported
17	Harper et al. (2013), United States	Cross-sectional	Yes	14 Adolescent medicine clinical care sites	HIV Stigma Scale—Negative self-image subscale; HIV-Positive Identity Questionnaire—Salience subscale	Internal consistency	HIV	200	16–24 (21.15, 1.91)/200	Sex: Male 100	Gay/queer 78; Bisexual 12; Straight 2.5; Trade 2.5; Down low 1.5; Questioning 1; Other 3.5	Ethnic identity: Black/African American 66.0; Hispanic/Latino 18.5; Non-Hispanic White 7.0; Native American 1.0; Asian American 0.5; Mixed race/other 7.0
18	Holmbeck & Kritikos (2022), United States	Longitudinal	Yes	3 hospitals and a statewide spina bifida association.	Extended Objective Measure of Ego Identity Status (EOM-EIS)—occupation and friendship domains	Inter-item correlation	Spina bifida (SB)	T7: 56	T7: 22–23 (22.71, 1:00)/56	Gender: T7-Male 55.4	Not reported	Ethnicity: White 82.35; Other 17.65
19	Hosek, Harper & Robinson (2002), United States	Cross sectional	Yes	Flyers distributed directly by clinicians	The Objective Measure of Ego Identity Status (OM-EIS	Internal consistency	HIV	8	17–21 (not reported)/8	Male 37.5, Female 62.5	Not reported	African-American 63; Latino 1(*n*); Multi-racial 1(*n*); Jamaican 1(*n*)
20	Ingersgaard et al. (2022), Denmark	Cross-sectional	Yes	Danish National Patient Registry (NPR).	Danish Illness Identity Questionnaire (IIQ-DK)	Internal consistency, Inter-item correlation, construct validity,	T1D	1170	15–26 (20.43, 3.09)^a^	Gender: Female 60.60	Not reported	Not reported
21	Lugasi et al. (2013), Canada	Cross-sectional	Yes	Diabetes clinics	The EOM-EIS-2	Internal consistency, Reliability	T1D; Renal transplant	T1D: 54; Renal transplant: 31	17.49–22.15 (T1D: 18.03; Renal transplant: 19.48)/85	Gender: Female 56; Male 44	Not reported	Cultural background: Caucasian 78; African American 2; Other 15
22	Luyckx et al. (2018), Netherlands	Cross-sectional	Yes	Refractory epilepsy: tertiary referral center in the Netherlands (Epilepsy Centre Kempenhaeghe) CHD: Belgian branch of Assessment of Patterns of Patient-Reported Outcomes in Adults with Congenital Heart disease—International Study (APPROACH-IS)	IIQ	Construct validity, internal consistency	Refractory epilepsy; CHD	Refractory epilepsy: 121; CHD: 191	18–40 (refractory epilepsy: 0.31, 6.50; CHD: 30.68, 5.03)^a^	Refractory epilepsy: Female 56.2; CHD: Female 49.5	Not reported	Not reported
23	Meyer & Lamash (2021), Isreal	Cross-sectional	Yes	Social media, celiac interest groups, Israel Celiac Rights Organization	IIQ	Internal consistency	Celiac disease	91	12–18 (14.67, 1.69)/34	Girls 72.5	Not reported	Not reported
24	Na et al. (2022), Belgium	Cross-sectional	No	Pediatric and congenital cardiology department of the University Hospitals Leuven (Belgium).	IIQ	Internal consistency, construct validity	CHD	255	24–28 (Median 26.3)^a^	Sex: Women 52.8	Not reported	Not reported
25	Oris et al. (2016), Belgium	Cross-sectional	Yes	Belgian Diabetes Registry	IIQ	Structural validity, internal consistency, construct validity	T1D	571	14–25 (18.9, 3.3)^a^	Men: 46.1; Women: 53.8	Not reported	Not reported
26	Oris et al. (2018), Belgium	Cross-sectional	Yes	CHD: database of congenital cardiology of the University Hospitals Leuven (Belgium); MSDs: database of rheumatology of the University Hospitals Leuven (Belgium)	IIQ	Structural validity, internal consistency, measurement invariance construct validity	CHD; multisystem connective tissue disorders (MSDs)	CHD: 276, MSDs: 241	CHD: 22–78 (36.8) MSDs: 17–81 (52.8)^a^	Sex: CHD: Men 54.3 MSDs: Men 17.4%	Not reported	Not reported
27	Peters and Brown (2022), United Kingdom	Cross-sectional	Yes	Participants were recruited online via email and social media channels.	IIQ	Internal consistency, construct validity	Inflammatory Bowel Disease	167	19–75 (39.8, 11.5)^a^	Gender: Female 78.4; Male 21.0; Other 0.6	Not reported	Not reported
28	Rassart et al. (2021), Belgium	Longitudinal	Yes	Belgian Diabetes Registry	IIQ	Internal consistency, construct validity	T1D	T1 = 559, T2 = 422, T3 = 381, T4 = 324 All = 276	14–25 (18.86, 3.25)^a^	T1—Girls 54%	Not reported	Not reported
29	Rassart et al. (2023), Belgium	Cross-sectional	No	IBD: Crohn’s & Colitis Foundation Belgium CHD: database of congenital cardiology of the University Hospitals Leuven Epilepsy: tertiary referral center in the Netherlands (Epilepsy Centre Kempenhaeghe) MSDs: database of rheumatology of the University Hospitals Leuven	IIQ	Structural validity, internal consistency, construct validity	Inflammatory Bowel Disease (IBD); CHD; Epilepsy; multisystem connective tissue disorders (MSDs)	IBD 109; CHD 213; Epilepsy: 116; MSDs 153	IBD : 18–60 (35.93, 10.96)^a^	Sex: IBD Men 23; Women 84	Not reported	Not reported
30	Rassart et al. (2012), Belgium	Longitudinal	Yes	Database of pediatric and congenital cardiology of the University Hospitals Leuven (Belgium) At T1, a control group was recruited at four secondary schools.	DIDS	Structural validity, internal consistency, construct validity	CHD	T1: 429	T1 14–18 (15.75, 1.14)^a^	Sex: T1—Boys 53.4	Not reported	Not reported
31	Raymaekers et al. (2020) , Belgium	Longitudinal	Yes	Belgian Diabetes Registry	IIQ	Internal consistency, construct validity	T1D	T1—545	14–25 (18.8, 3.2)^a^	T1—Girls 54.1	Not reported	Not reported
32	Shani and Van Zalk (2024), Germany	Cross-sectional	No	Youth Committee of the German Celiac Society (DZG)	IIQ —Acceptance and Enrichment	Structural validity, internal consistency, construct validity	Celiac disease	165	14–22 (17.18, 2.50)^a^	Gender: Male 17; Female 81; Undisclosed 2	Not reported	Not reported
33	Stepleman et al. (2017), United States	Cross-sectional	Yes	Multiple Sclerosis Center in the southeastern United States.	Identity Reconstruction Assessment Scales (IRAS)	Structural validity, internal consistency, construct validity	MS	137	20–74 (45.50, 10.76)^a^	Female 84.67	Not reported	Race/ethnicity: Native American 0.7; African American 39.4; Hispanic/Latino 4.4; Caucasian 55.6
34	Van Bulck et al. (2021),Belgium	Longitudinal	No	Data of the Belgian branch of the APPROACH-IS project (Apers et al., 2015)	IIQ	Internal consistency, construct validity	CHD	276	T1—22–78 (34)^a^	T1—Men 54; Women 46	Not reported	Not reported
35	Van Bulck et al. (2018), Belgium	Longitudinal	Yes	Data of the Belgian branch of the APPROACH-IS project (Apers et al., 2015)	IIQ	Internal consistency, construct validity	CHD	T2014: 216	T2014 23–79 (35)^a^	T2014—Men 49.5	Not reported	Not reported
36	Vanderhaegen et al. (2024), Belgium	Longitudinal	Yes	Belgian Diabetes Registry	DIDS; IIQ	Internal consistency, construct validity	T1D	T1—558	T1—14–25 (18.85, 2.34)^a^	T1—Female 54	Not reported	Ethnicity: Belgian 98; Dutch 1.3; Others 0.7
37	Verschueren et al. (2020), Belgium	Cross-sectional	Yes	Belgian Diabetes Registry	DIDS	Structural validity, internal consistency, construct validity	T1D	431	14–25 (18.89, 3.25)^a^	Gender: Male 47.1; Female 53.8	Not reported	Not reported

a Indicates not separated in reporting.

Across all papers, 9,486 participants were included with an age range of 13–83 years. However, only five papers provided separate data for participants in the age range 16–24 years, representing 383 participants from the overall sample. Seven papers were longitudinal, with the remaining employing a cross-sectional design. Papers were mainly based in Belgium (*n* = 12) and the United States (*n* = 10).

Table 3 presents the definitions of identity presented in the included papers. In reviewing the definitions of identity across the included papers, 12 did not provide an explicit definition of identity (^01,02,03,05,06,10,12,15,24,29,32,34^), with one using the term identity interchangeably with self-concept (^15^). The remaining papers offered different definitions of identity, as summarized below. Eleven papers explicitly referenced Erikson’s (1968) theory of identity development (^13,14,19,21,26,28,30,31,36,37^), highlighting identity development as a process involving crisis, exploration, and commitment. Five papers (^04,07,08,09,16^) conceptualized identity as a sense of continuity, stability, and uniqueness across time and life changes, most commonly citing Bosma and Kunnen (2001). Six papers (^07,11,21,22,27,34^) adopted a lifespan approach to explaining identity, and its dependence on social and contextual factors. Eight papers focused specifically on adolescence and emerging adulthood as critical periods for identity exploration across multiple life domains, emphasizing identity as domain-specific and evolving through exploration (^14,17,18,19,20,21,23,25^). Additionally, two papers framed identity as multidimensional, encompassing various elements of the self (^11,35^).

**Table 3.** Definitions of identity provided in included papers.^a^

Code	Author (year)	Definition of identity
04	April et al. (2022)	“Identity is often thought of as a singular concept describing a collective aspect of who we are.”
07	Calandri et al. (2019)	“…defined by psychological literature as the sense of continuity and oneness which everyone experiments during one’s life, in spite of the continuous changes in their biological, psychological, and social lives.”
08	Calandri et al. (2018)	“Identity is the sense of continuity and oneness that everyone experiences during one’s life despite the continuous biological, psychological, and social changes (Bosma and Kunnen, 2001).”
09	Calandri et al. (2020)	“Identity has been defined as the sense of uniqueness and continuity over time that everyone experiences during one’s life despite the continuous changes in biological, psychological, and social aspects of life (Bosma and Kunnen, 2001).”
11	Commissariat et al. (2023)	“Identity is a framework housing one’s self-concept, self-esteem, relationship roles, values and future potential; it is further shaped by supplementary influences such as environments, personal experiences, expectations and perceptions of others.”
13	Gavaghan & Roach (1987)	“According to Erikson (1968), adolescents achieve a sense of identity by going through “crisis” periods in which they explore and question values, beliefs, and goals previously accepted from parents and others. They then develop and make commitments to their own personal set of goals, values, and beliefs”
14	Geuens et al. (2023)	“Adolescence and emerging adulthood are two such critical phases, during which youth must work on their identity development (Arnett, 2000; Erikson, 1968) Achieving a strong identity is considered a daunting challenge for contemporary youth (Arnett, 2000; Luyckx & Seiffge-Krenke, 2009; Oris et al., 2016).”
16	Graziano et al. (2020)	“Identity is the sense of uniqueness and continuity over time that everyone experiences during one’s life despite the continuous changes in biological, psychological, and social aspects of life (Bosma and Kunnen, 2001).”
17	Harper et al. (2013)	“The formation of an individualized identity is considered by many theorists to be the primary developmental goal of the adolescent years.”
18	Holmbeck & Kritikos (2022)	“In addition, emerging adulthood is a critical period of identity development with youth exploring and experimenting with different options across different functioning domains (e.g., occupational and social relationships)”
19	Hosek, Harper & Robinson (2002)	“The formation of a sense of identity is considered by many theorists to be the primary developmental goal of the adolescent years (Adams et al., 1992). Erikson (1950) described adolescence as the period in the lifespan during which an individual must establish a sense of personal identity and avoid the dangers of identity diffusion and role confusion.”
20	Ingersgaard et al. (2022)	“Key developmental processes of identity formation take place during adolescence and emerging adulthood.”
21	Lugasi et al. (2013)	“Adolescence is a period during which the individual experiences a crisis that is resolved by making commitments concerning the future in the domains of occupation, religion, and ideology. Identity development does not take place in a vacuum and can be greatly influenced by the personal and social context in which individuals find themselves. Many researchers and clinicians consider the central task of adolescence to be identity formation, well delineated by Erikson’s theory of identity development”
22	Luyckx et al. (2018)	“Identity constitutes a developmental construct that changes through the life-span, with most identity changes occurring in adolescence and the transition to adulthood.”
23	Meyer & Lamash (2021)	“Developing identity is a central developmental stage during adolescence and young adulthood. It shapes and is shaped by relationships with others, guides daily behaviors and choice-making, and is closely associated with well-being”
25	Oris et al. (2016)	“Identity development constitutes a core developmental task during adolescence that may well extend into the late teens and twenties (a period known as emerging adulthood), due to the postponement of adult role attainment in current postmodern societies.”
26	Oris et al. (2018)	“Inspired by Erikson’s (1968) seminal work on lifespan ego development, identity is viewed as the degree to which (i) an individual (manages to) integrates different self-assets into a coherent sense of self, and (ii) such a coherent sense of self translates itself into daily life and guides choices and values.”
27	Peters & Brown (2022)	“Identity Theory defines personal identity as a set of meanings that characterizes the self in terms of group membership, social roles, and personal characteristics (Burke & Stets, 2009). As such, an individual possesses multiple self-concepts that are interrelated to form an overall identity (Burke & Stets, 2009).”
28	Rassart et al. (2021)	“One such developmental challenge is building a sense of identity, which requires young persons to integrate different self-assets into a coherent sense of self and commit to important life choices and goals (Erikson, 1968).”
30	Rassart et al. (2012)	“An important developmental task for late adolescents is personal identity formation. Based on Erikson [1] and Marcia [2], Luyckx and colleagues have proposed an identity model that includes both the formation and the evaluation of identity commitments.”
31	Raymaekers et al. (2020)	“Identity formation is an important developmental task for adolescents in industrialized nations. While coping with rapid hormonal and bodily changes, they are confronted with life questions such as ‘Who am I?’ and ‘Who do I want to become?’ (Erikson, 1968).”
33	Stepleman et al. (2017)	“Identity is a conglomeration and expression of people’s stories about themselves and their connections to others that tie together differing aspects of their self-concept. It is a socially concocted and construed understanding of who one is, predicated upon how one came to be that way and with implications upon one’s aspirations for the future (Dunn & Burcaw, 2013)”
35	Van Bulck et al. (2018)	“Identity can be defined as the degree to which an individual integrates different self-assets into a coherent sense of self, which guides choices and values in daily life.”
36	Vanderhaegen et al. (2024)	“Identity formation is a lifelong process but is particularly salient during adolescence and emerging adulthood (Erikson, 1968).”
37	Verschueren et al. (2020)	“According to Erikson (1968), the task of identity formation is a continuous and lifelong process that peaks in adolescence and emerging adulthood. In these age periods, individuals are expected to make personal life choices and attain a set of self-identified values and goals, indicative of identity synthesis.”

a Only included papers that explicitly provided a definition of identity are shown.

### Overview of measure characteristics

Table 4 presents an overview of measures included in this review. A wide variety of theoretical concepts and frameworks informed the development of measure items. Five measures (Ego Identity Incomplete Sentence Blank [EI-ISB], Extended Objective Measure of Ego Identity Status [EOM-EIS], Ego Identity Process Questionnaire [EIPQ], EOM-EIS-2, and Objective Measure of Ego Identity Status [OM-EIS]) drew on Ego Identity Status theory (Erikson, 1968; Marcia, 1966), while two (Illness Identity Questionnaire [IIQ] and Accepting Diabetes and Personal Treatment [ADAPT]) were informed by Illness Identity Theory (Charmaz, 1995; Oris et al., 2018). The centrality of stigmatizing attributes to identity was explored in two measures (Collective Self-Esteem Scale [CSS] and HIV Centrality), based on work by Quinn and Chaudoir (2009). One measure (DIDS) reflected the five dimensions of identity development presented by Luyckx et al. (2008), and another (Identity Reconstruction Assessment Scales [IRAS]) drew from qualitative research on identity reconstruction (Yoshida, 1993). Additional measures were informed by extensive review of stigma literature and clinical expertise (HIV Stigma Scale [HSS]), the Motivated Identity Construction Theory (Identity Motives Scale [IMS]; Vignoles et al., 2006), and Social Identity Theory (CSS; Tajfel & Turner, 1979). Others incorporated broader theoretical explorations of identity and self-concept (HIV-Positive Identity Questionnaire [HPIQ]), identity change across four domains (Identity change: amount and affective change), and mixed-method research on HIV-related identity changes (Impact on Self Concept Scale [ISCS]; Updegraff et al., 2002).

**Table 4.** Characteristics of identity development measures (original development studies).

Measure (author/s)	Primary purpose	Theory/constructs captured	Target population	Number of items (subscales)	Scoring information	Language (translations)	LTC-P observed in review (*n*)
Illness Identity Questionnaire (IIQ) (Oris et al., 2016)	To assess the concept of illness identity, or the degree to which Type 1 diabetes is integrated into one’s identity.	Illness Identity (Charmaz, 1995; Oris et al., 2016), and the domains of engulfment; rejection; acceptance; and enrichment	Age: 14–25 years Sample: Type 1 diabetes	25 (Engulfment 8 items; Rejection 5 items; Acceptance 5 items; and Enrichment 7 items.)	5-point Likert scale: 1 (*strongly disagree*) to 5 (*strongly agree*). Mean scores can be calculated for four illness identity dimensions.	Dutch (English; Danish; German; Chinese (modified), Hebrew)	Type 1 diabetes (3,081); Congenital Heart Disease (1,885); Multisystem connective tissue disorders (394); Inflammatory bowel disease (276); Mayer–Rokitansky–Kuster–Hauser syndrome (263); Celiac disease (256); Epilepsy (237); Neuromuscular disorders (118)
Dimensions of Identity Development Scale—DIDS (Luyckx et al., 2008)	To assess core dimensions of identity development.	Five dimensions of identity development (Luyckx et al., 2008): Commitment making; Exploration in breadth; Ruminative exploration; Identification with commitment; Exploration in depth	Age: 17–20 years Sample: Normative	25 (Commitment making 5 items, Exploration in breadth 5 items: Ruminative exploration 5 items, Identification with commitment 5 items, Exploration in depth 5 items)	5-point Likert type rating scale: 1 (*strongly disagree*) to 5 (*strongly agree*). Mean scores can be calculated for each five dimensions of identity.	Dutch (English; Polish (modified); Greek; Finnish)	Type 1 diabetes (989); Long QT Syndrome (6); Hypertrophic cardiomyopathy (5); Dilated Cardiomyopathy (1); Congenital Heart Disease (429)
Identity Motives Scale—IMS (Vignoles et al., 2006)	To assess the six identity motives of the Motivated Identity Construction Theory	Motivated Identity Construction Theory (Vignoles et al., 2006): self-esteem, efficacy, continuity, belonging, distinctiveness, and meaning.	Age: 19–51 years Sample: Normative	12	5-point Likert scale: 1 (*extremely disagree*) to 5 (*extremely agree*) with negative items reverse coded. Higher scores on each subscale represent higher satisfaction of each identity motive.	English	Multiple Sclerosis (331)
Accepting Diabetes and Personal Treatment—ADAPT (Commissariat et al., 2023)	To assess how adolescents and young adults with Type 1 diabetes (T1D) incorporate their condition into their identity	Illness identity (Charmaz, 1995; Oris et al., 2016): Stigma management, Adjustment to Perceived Interference, Benefit-finding	Age: 13–25 years Sample: Type 1 diabetes	18 (Stigma management 6 items, Adjustment to perceived interference 6 items, benefit-finding 6 items)	5-point Likert scale: 0 (*strongly disagree*) to 4 (*strongly agree*). All items are scored on a 0–4 scale, with higher scores indicating more positive stigma management, more adjustment to perceived interference, and more benefit-finding practices.	English	Type 1 diabetes (165)
Modified version of the identity subscale of the Collective Self-Esteem Scale—CSS (Earnshaw et al., 2015)	To assess extent to which HIV is a central aspect of an individual’s identity.	Social identity theory (Tajfel & Turner, 1979); Centrality of stigmatizing attributes to identity (Quinn & Chaudoir, 2009).	Age: 21–63 years Sample: HIV	4 (two adapted from the original identity subscale and two based on previous work with people living with concealable stigmatized identities)	5-point Likert scale: 1 (*strongly disagree*) to 5 (*strongly agree*). A composite score is created by averaging items.	English	HIV (93)
Ego Identity Incomplete Sentence Blank—EI-ISB (Marcia, 1966)	To assess overall ego achievement.	Ego Identity Status (Marcia, 1966)	Age: 18+ years Sample: Normative; Male	23	Sentences are completed by participant and scored on a 3-point scale, based on standard scoring procedures for the degree of identity achievement demonstrated. An Ego Identity score represents the sum of the ratings for the 23 sentences.	English	Cancer (42)
Extended Objective Measure of Ego Identity Status—EOM-EIS (Grotevant & Adams, 1984)—subscales	To assess four identity statuses: Identity Achievement, Moratorium, Diffusion and Foreclosure.	Ego Identity Status (Marcia, 1966)	Age: 18+ years Sample: Normative	64	5-point Likert scale: 1 (*strongly disagree*) to 6 (*strongly agree*). Following these 64 items, respondents rate each of the eight domains in terms of “how actively you are thinking about these issues in your life at the present time” on a 4-point Likert scale: 1 (*not dealing with this issue at all now*) to 4 (*dealing with this area to a very strong degree*).	English	Spina bifida (68)
HIV Centrality (Brener et al., 2013)	To assess the centrality of HIV to identity	Centrality of stigmatizing attributes to identity (Quinn & Chaudoir, 2009).	Age: 18–77 years Sample: HIV	1	11-point scale: 0 (*HIV is not a strong part of how they saw themselves*) to 10 (*HIV is a very strong part of how they saw themselves*)	English	HIV (181)
HIV Stigma Scale (HSS) (Berger et al., 2001)—Negative Self-Image subscale	To measure stigma perceived by people with HIV	Conceptual model of perceived stigma based on extensive review of stigma literature, supplemented by clinical experts.	Age: 19–82 years Sample: HIV	40	4-point Likert-type scale: (*strongly disagree*, *disagree*, *agree*, and *strongly agree*).	English	HIV (200)
HIV-Positive Identity Questionnaire (HPIQ) (Carter, 2005)—Salience subscale	To measure the extent to which, and how positively or negatively, individuals have integrated being HIV-positive into their self-concept.	Theoretical exploration of construct of identity, to assess reflexive self-concept, or “felt” identity.	Age: 40 years (mean) Sample: HIV	18	5-point Likert-type scale: (“*strongly disagree*” to “*strongly agree*”)	English	HIV (200)
Identity change: amount and affective change (April et al., 2022)	To measure the perceived amount of identity change, and perceived affective change for each aspect of identity.	Four identity dimensions (Carter, 2017): personal, social, relational and performative.	Age: 19–73 years Sample: Chronic illness	8 (4 amount, 4 affective)	Amount: Participants are asked to think about who they are since diagnosis; then rate how much they have changed as a person (personal identity), in terms of identifying with social groups (social identity), who they are in their relationships (relational identity), and how they act or communicate about themselves (performative identity). Each item was measured on a scale from 1 (*not at all*) to 7 (*very much*). Affective: Participants asked to assess whether they viewed change in that identity aspect since their diagnosis to be negative, neutral, or positive. Items measured on a 7-point scale: range 1 to 7, where a value of 4 indicated a change that was perceived as neither more negative nor more positive, values <4 a negative change, values >4 a positive change.	English	Type 1 diabetes (345)
Identity Reconstruction Assessment Scales—IRAS (Stepleman et al., 2017)	To assess identity reconstruction in patients with multiple sclerosis.	Qualitative work (Yoshida, 1993) describing a “pendular” dynamic concept of identity reconstruction (former self, supernormal identity, middle self, disabled identity as an aspect of total self, and disabled identity as total self).	Age: 20–74 years Sample: Multiple sclerosis	23 (Sustained identity 9 items, Reactionary identity 7 items, Integrated Identity 7 items)	6-point rating scale: 1 (*strongly disagree*) to 6 (*strongly agree*). Higher scores reflect greater alignment with the particular identity classification being measured (e.g., higher scores on items reflecting “former self” reflect greater identification with the former self).	English	Multiple Sclerosis (137)
Impact on Self Concept Scale—ISCS (Golub et al., 2013)	To measure identity-related growth and loss in HIV-positive gay and bisexual men	Examining positive and negative HIV-related identity changes, self-growth and self-loss (Updegraff et al., 2002)	Age; 18–54 years Sample: HIV; Male	10	Participants were asked to respond to both positive and negative items by indicating how often the feel similarly to each statement, using a scale ranging from 1 (*never*) to 6 (*always*).	English	HIV (129)
The Ego Identity Process Questionnaire—EIPQ (Balistreri et al., 1995)	To assess the ego identity dimensions of exploration and commitment.	Ego Identity Status (Marcia, 1966)	Age: 17–24 years Sample: Normative	32	6-point Likert-type scale: 1 (*strongly disagree*) to 6 (*strongly agree*). Scoring is reversed for negatively-stated items. Item scores are summed to obtain total scores for exploration and commitment separately, each of which can range from 16 to 96.	English	Epilepsy (51)
The EOM-EIS-2 (Bennion & Adams, 1986)	To assess Ideological and Interpersonal ego identity status	Ego Identity Status (Marcia, 1966)	Age: 18–45 years Sample: Normative	64	6-point Likert scale: A (*strongly agree*) to F (*strongly disagree*).	English	Type 1 diabetes (54); Renal transplant (31)
Objective measure of ego identity status—OM-EIS (Adams et al., 1979)	To assess ego identity status	Ego Identity Status (Marcia, 1966)	Age: 17-51 years Sample: Normative	24 (Diffusion 6 items, Foreclosure 6 items, Moratorium 6 items, Identity achievement 6 items)	6-point Likert scale: (*strongly agree* to *strongly disagree*)	English	HIV (8)

The study samples of the original measure development papers varied from adolescents, young adults, and older adults (range 13–82 years). Eight measures were developed for use with a normative population (DIDS, IMS, EI-ISB, EOM-EIS, EIPQ, EOM-EIS-2, and OM-EIS), five were specifically developed for individuals with HIV (ISCS, HPIQ, HSS, HIV Centrality, and CSS), one was developed for use with any chronic illness (Identity change: amount and affective change), one for those diagnosed with Type 1 diabetes (ADAPT), and one for MS (IRAS). Most measures were developed in English, although several have been translated into other languages (e.g., Dutch and German).

Across all papers identified for review, only three measures were used in more than one paper: 18 used the IIQ (Oris et al., 2016), 4 used the Dimensions of Identity Development Scale (DIDS; Luyckx et al., 2005), and 4 used IMS (Vignoles et al., 2006). The remaining 15 measures were used in a single study each.

### Quality of design, methodology, and reporting

Using Cohen et al.s’ (2008) criteria for evaluating evidence-based assessment, only three measures were rated as “well-established,” with the remaining measures rated as “promising.” Quality was only assessed for these three, as the authors considered that analysis would only be meaningful where data were available from more than one study. Consequently, only the IIQ (Oris et al., 2016), DIDS (Luyckx et al., 2005), and IMS (Vignoles et al., 2006) were assessed for quality using an adapted COSMIN checklist version 1.0 (Mokkink et al., 2018). Table 5 shows a summary of the quality assessment, and full COSMIN evaluations can be seen in online supplementary material 3. The content validity of all papers was synthesized using a narrative review and will be represented at the end of this section.

**Table 5.** Quality assessment using COSMIN by measure.

	IIQ		DIDS		IMS	
	**Overall rating +/−/?**	**Quality of evidence** ^a^	**Overall rating +/−/?**	**Quality of evidence** ^a^	**Overall rating +/−/?**	**Quality of evidence** ^a^
Structural validity	+	High	+	High		
Internal consistency	+	High	+	High	+	Low
Measurement invariance	?	Moderate				
Construct validity	+	High	+	High	+	Moderate
**COSMIN category**	A		A		B	

*Note*. In the COSMIN evaluation, “+” indicates sufficient assessment and “?” indicates indeterminate assessments. Empty cells in the table indicate that none of the included studies provided a relevant analysis for a measurement property. IIQ = Illness Identity Questionnaire; DIDS, Dimensions of Identity Development Scale; IMS, Identity Motives Scale.

a Quality of evidence ratings: High, moderate, low, very low.

The included papers only rated four measurement properties—structural validity, internal consistency, measurement invariance, and construct validity. Additionally, COSMIN requires the review team to create a hypothesis for testing construct validity; however, due to the diverse nature of constructs and comparator instruments being captured in this review, we chose to only assess construct validity with regard to the hypothesis chosen by the authors of the studies.

#### Risk of bias: quality assessment of methodology

Only the measurement properties reported in the included papers are presented below; properties not shown were not assessed in those papers.

The IIQ received a “Very good” risk of bias rating for structural validity, internal consistency, and construct validity. Only one study (Oris et al., 2018) looked at measurement invariance, receiving an “Adequate” score.

The DIDS received a “Very good” rating for structural validity, internal consistency, and construct validity.

The IMS received a “Very good” rating for construct validity. It received a “Doubtful” rating for internal consistency due to papers reporting six subscales that contributed to a total score, but only providing a single Cronbach’s Alpha, making it unclear whether the subscales could be reliably interpreted on their own.

#### Overall ratings of psychometric properties of measures

The IIQ received a “Sufficient” rating for structural validity, internal consistency, and construct validity. Only one study (Oris et al., 2018) looked at measurement invariance, receiving an “Indeterminate” score due to no multiple group factor analysis or differential item functioning analysis performed.

The DIDS received a “Sufficient” rating for structural validity, internal consistency, and construct validity.

The IMS received a “Sufficient” rating for internal consistency and construct validity.

### GRADE evidence

The quality of evidence GRADE ratings was mostly influenced by risk of bias and indirectness. Structural validity was of “high” quality evidence across the IIQ and DIDS. The IMS did not report any structural validity assessments. Internal consistency for the IIQ and DIDS was “High,” whereas the IMS scored “Low” due to serious risk of bias (multiple studies of doubtful quality) and serious indirectness. The rationale for the risk of indirectness was that IMS studies included in the review were performed in populations aged 10–57 years, with averages ranging from 25 to 38 years; this was deemed to be indirect as the current review is interested in those aged 16–24 years. The one assessment of measurement invariance for IIQ was “Moderate” quality due to a serious risk of bias (only one study of adequate quality). Quality of evidence for construct validity in the IIQ and DIDS was “High.” The IMS scored “Moderate” on construct validity due to serious indirectness for the reasons noted previously.

### Narrative review of content validity

The content validity for all 16 measures, using the original measure development study or the study in which a measure had been heavily modified, as well as any further content validity performed in the review papers, was assessed by one reviewer (T.R.). One measure, the IMS, could not be assessed because the original referenced development papers (Manzi et al., 2010; Vignoles et al., 2006) did not clearly explain how the content aligned with the measure manual reviewed by the assessors and did not offer any validation information. Additional content validity of the IIQ was performed in two papers that reported translations of the measure into Danish and Hebrew (Ingersgaard et al., 2022; Meyer & Lamash, 2021).

Full details of content validity are presented in Table 6. Across the reviewed measures, expert involvement was often not reported. In the few instances where involvement was described, experts contributed through activities such as revising items based on clinical experience or providing specialized input during the development process. However, the specific roles, qualifications, and expertise of the experts involved were rarely detailed. As a result, while some engagement was reported, there appeared to be limited systematic engagement with professional expertise throughout the reviewed measures.

**Table 6.** Content validity processes across measures.

Measure (References)	Concept elicitation	Item generation	Professional/expert involvement	Lived experience involvement
IIQ (Ingersgaard et al., 2022^a^; Meyer & Lamash, 2021^a^; Oris et al., 2016)	Theory-driven	Original development study: Items from existing measures, as well as newly generated items based on measures. Theory-driven.	Original development study: Not reported Validation of Danish version: Two experts (of Social Science and diabetes) assessed relevance and comprehensiveness regarding cultural appropriateness of IIQ and found items matched English version and theoretical constructs. Validation of Hebrew version: Face validity with five experts in child development and chronic health conditions. Reviewed questionnaire for content and understandability found no major issues.	Original development study: Not reported Validation of Danish version: Relevance and intelligibility of the items, responses, and instructions assessed using cognitive interviews with 16–26-year-olds with Type 1 diabetes. Led to linguistic modification of two items. Validation of Hebrew version: Face validity with 5 adolescents and young adults with celiac disease. Reviewed questionnaire for content and understandability found no major issues.
DIDS^b^ (Luyckx et al., 2008)	Theory-driven	Items on four identity dimensions revised from existing measures, new items generated to assess ruminative exploration. Theory-driven.	Not reported	Not reported
ADAPT (Commissariat et al., 2023)	Theory-driven	Based upon previous qualitative and mixed-methods research by authors, and previous illness identity literature.	Items revised based on clinical experience by study team.	Cognitive debriefing performed with two youth with Type 1 diabetes, resulting in language revisions.
Modified version Collective Self Esteem Scale, identity subscale (Earnshaw et al., 2015)	Adapted from existing scale	Two items adapted from existing scale, two based on work with people living with stigmatized identities.	Not reported	Not reported
EI-ISB^b^ (Marcia, 1966)	Theory-driven	Based on Erikson’s theory of ego identity.	Not reported	Not reported
EOM-EIS^b^ (Grotevant & Adams, 1984)	Theory-driven	Based on ego identity theory	Not reported	10 graduate students read descriptions of the identity statuses and then judged which category was appropriate for each item. The overall mean percentage agreement across the 10 raters for the 64 items was 96.5%.
HIV Centrality (Brener et al., 2013)	Centrality of stigmatizing attribute to identity—Quinn & Chaudoir (2009)	One item directly related to HIV centrality.	Not reported	Not reported
HIV Stigma Scale (Berger et al., 2001)	Conceptual model of perceived stigma created for development, based on extensive review of literature.	Item pool based on conceptual model.	Seven expert content reviewers provided with conceptual model and asked to judge relevance, clarity and comprehensiveness of items. Experts also asked to generate additional items.	Pretesting with nine people with HIV. Asked for feedback on items that were confusing or irritating. Interviewed to identify any issues with items including change of mood when completing measure.
HIVPIQ (Carter, 2005^c^)	Theory-driven	Adapted from other measures of identity or created by author and content reviewers using reviews of literature. Pilot study.	Six expert content reviewers provided with rationale and explanation of supporting theory, assessed each item for applicability, clarity and appropriateness to constructs and sub-scales. Experts also asked to generate additional items.	Focus group of three adolescents living with HIV (although more were planned). Relatedness assessed using construct/item matching exercise and open-response questions. Sample size too small for analysis.
Identity change (April et al., 2022)	Theory-driven, and extensive qualitative research	Based on existing research.	Not reported	Not reported
IRAS (Stepleman et al., 2017)	Conceptual model based upon existing qualitative research.	Study team reviewed the five reconstructed identity categories in conceptual model, and created items for each.	Not reported	Not reported
ISCS (Golub et al., 2013)	Based upon existing mixed-methods research.	Iterative free-listing approach using underlying constructs.	Small group of key informants (details not specified, unsure if professional/expert or lived-experience involvement) vetted items then grouped them into categories designed to be theoretically meaningful for the constructs of self-growth and self-loss.	
EIPQ^b^ (Balistreri et al., 1995)	Theory-driven	Pilot study conducted to generate items. Items from existing measures revised.	Five graduate students in psychology given descriptions of dimensions, and then rated which item belonged to which dimension. Items with less than 80% agreement were discarded.	Not reported
EOM-EIS-2^b^ (Bennion & Adams, 1986)	Theory-driven	Ambiguous items from EOM-EIS rewritten to indicate presence or absence of a crisis period and show degree of commitment.	Not reported	Nine college students read descriptions of the four identity statuses and then judged which category was appropriate for each item. The overall percentage of agreement among nine raters was 94.4%.
OM-EIS^b^ (Adams et al., 1979)	Theory-driven	Items constructed based on ego-identity theory. Brief pilot study led to revision of items.	Not reported	Not reported

*Note*. IIQ = Illness Identity Questionnaire; DIDS, Dimensions of Identity Development Scale; EI-ISB = Ego Identity Incomplete Sentence Blank; EOM-EIS = Extended Objective Measure of Ego Identity Status; PIQ = Positive Identity Questionnaire; IRAS = Identity Reconstruction Assessment Scales; ISCS = Impact on Self Concept Scale; EIPQ = Ego Identity Process Questionnaire; OM-EIS = Objective Measure of Ego Identity Status.

a Translation studies included in review.

b Developed and validated with normative sample.

c Not peer reviewed.

Similarly, lived experience involvement was frequently not reported. Where it was described, involvement typically took the form of feedback sessions with individuals who had relevant lived experiences; one measure specifically noted the use of cognitive debriefing (Commissariat et al., 2023). These contributions were intended to enhance the relevance, clarity, and acceptability of the measure items. Nevertheless, comprehensive reporting on the nature and integration of lived experience feedback was limited, again suggesting systematic and meaningful engagement with lived experience perspectives was not consistently prioritized across the measures.

### COSMIN category of measures

Using an adapted COSMIN categorization, the IIQ and DIDS were placed in Category A (at least low-level quality evidence for sufficient internal consistency) and thus can be tentatively recommended for use in AYA with LTC-P. An important caveat in this recommendation is the need for content validity studies, using expert and lived experience voices, to be undertaken to ensure all measures adequately reflect the construct in the selected study population. While the IMS did demonstrate low-level evidence for sufficient internal consistency as required for Category A, the review team felt the lack of content validity was sufficient to downgrade the level to Category B, requiring further research to assess the quality of the measure for use in AYA with LTC-P.

## Discussion

We identified 37 papers reporting the use of measures of identity with individuals living with LTC-P aged 16–24 years. Across these studies, 16 unique self-report measures were used. Evaluation of the psychometric properties and methodological quality of the measures highlighted both strengths and limitations, offering valuable insights into how identity has been assessed in this population and how it could be measured in future research and clinical practice.

### Recommendations

When selecting a measure, users should carefully consider the relevance of each instrument to their specific aims. The IIQ may be suitable when examining illness identity, but it may not capture the broader reconstruction and negotiation of identity for AYA following a diagnosis of LTC-P. Similarly, the DIDS assesses an individual’s stage of personal identity development and does not specifically address issues related to living with an LTC-P, such as the reaction to disrupted life trajectories (Hale et al., 2015; Kirk & Hinton, 2019) and shifts in social roles (Harris et al., 2003; Packham et al., 2020). Neither measure was originally co-produced with experts or young people with lived experience, ensuring content validity. Although later translations of the IIQ incorporated limited content validity testing for cultural and linguistic reasons (Ingersgaard et al., 2022; Meyer & Lamash, 2021), future use, especially in unvalidated populations, should include a systematic evaluation of content validity to ensure appropriateness. The remaining 13 measures identified in this review demonstrated promising assessment (Cohen et al., 2008). However, future validation studies are recommended before their widespread use, particularly for measures developed some time ago, and/or within normative samples, as their content may not be fully applicable to contemporary or specific populations.

### Limitations of measures

A consistent limitation of measures included in this review was the lack of systematic assessment of content validity, particularly for measures originally developed for use in normative populations (e.g., “healthy” adolescents). Most measures were developed by applying theoretical frameworks (often developed for adult or normative populations) or by adapting items from existing measures, with only limited involvement from experts or individuals with lived experience. This gap in content validity risks measurement error and could limit clinical usefulness in these populations. Notably, the ADAPT measure (Commissariat et al., 2023), developed for individuals with Type 1 diabetes, was the only tool to include cognitive debriefing and represented the most comprehensive approach to content validation identified in this review. To enhance validity and relevance, future measures should integrate input from both experts and AYA with lived experience through structured participatory methods (e.g., focus groups, Delphi studies, interviews, cognitive interviewing, and pilot testing) and report these processes transparently.

Many of the measures included in this review were developed within the framework of developmental psychology, often drawing on Eriksonian theories of identity formation. While these theoretical models offer valuable insights into normative identity development across the lifespan, they may not fully capture the unique challenges faced by AYA living with LTC-P. For example, identity processes in this population may be shaped by factors such as disrupted life trajectories (Hale et al., 2015; Kirk & Hinton, 2019) and shifts in social roles (Harris et al., 2003; Packham et al., 2020), experiences not typically reported in measures grounded in normative developmental theory. As such, the direct application of these tools to clinical populations should be approached with caution, and further validation work is needed to determine their relevance to the lived experience of those with LTC-P. It is worth noting that some measures included in this review (April et al., 2022; Golub et al., 2013; Stepleman et al., 2017) explicitly address aspects of identity reconstruction, change, and growth in individuals living with LTC-P. These constructs may have important clinical relevance and could inform the development of targeted psychosocial interventions.

### Limitations of papers

A further consideration identified in this review was the variation in how identity was conceptualized across the included studies. Although this review does not favor one definition of identity over another, it emphasizes the importance of conceptual clarity. While variation in theoretical framing is not inherently problematic, different conceptualizations can offer valuable perspectives depending on research aims, it is essential that authors clearly articulate their definition and/or theoretical model/s of identity. However, many papers failed to provide an explicit conceptualization of identity, making it difficult to assess the appropriateness of the measures used or compare results across papers. The lack of clarity around the construct aiming to be measured introduces ambiguity and undermines the validity and utility of the research. Clear communication of how identity is defined and understood is therefore critical, particularly when assessing populations whose identity development may differ from normative patterns.

Additionally, across the included studies, reporting of sociodemographic characteristics was often limited or inconsistent. Data on religion were entirely absent, and information on race and ethnicity was sparse and, on occasion, conflated into a single category. Reporting of sexual orientation was similarly limited (only four papers reported this), and data related to sex and gender were frequently reported interchangeably, despite representing distinct constructs. In several studies, sex and/or gender were reported only for a single category (e.g., percentage female), which meant it was not possible to determine if any transgender, intersex, or non-binary AYA were included in the sample. Socioeconomic status was most commonly reported on via educational attainment; however, this is of limited relevance for the AYA cohort considered in this review, as many AYA are likely to still be in education, to experience educational delays, or be in transition between educational stages. These limitations restrict the extent to which experiences of identity development among marginalized groups can be examined and highlights the need for future research to adapt more comprehensive and conceptually robust approaches to collection and reporting of sociodemographic data.

### Strengths and limitations of this review

This review provides the first systematic synthesis of identity-related measurement tools that have been used with AYA living with LTC-P. Adhering to COSMIN methodology, this review has produced a comprehensive and methodologically rigorous evaluation of the psychometric properties of included measures.

Several limitations should be acknowledged. First, the use of the COSMIN framework was applied in full to only three measures, as analysis would only be meaningful where data were available from more than one study. As a result, many of the measures identified remain in need of further psychometric evaluation.

Additionally, COSMIN’s stringent criteria, while promoting high standards in measure development, have been criticized as overly conservative (Rothmund et al., 2023; Smith et al., 2021). As many measures were developed prior to the publication of key methodological guidelines, they may have been undervalued in this review. Additionally, the reliance on COSMIN’s lowest-rating counts scoring approach may have overlooked more nuanced differences in methodological quality across studies. Amendments were made to COSMIN scoring when necessary, e.g., by accepting evidence of factor structure from other studies to support internal consistency. The content validity assessment was conducted narratively, due to COSMIN’s requirement for both lived experience and expert involvement in measure development for any rating above “low quality.” Similarly, due to the diversity in constructs assessed across measures, it was not feasible to generate consistent hypotheses for construct validity testing, leading to an “indeterminate” rating for many studies that did not explicitly propose one. These adaptations, while necessary, may have underestimated some measures’ strengths.

Further limitations relate to the scope and design of the review. Despite efforts to broaden the search strategy, incomplete reporting, inability to contact authors, and exclusion of non-English papers may have introduced cultural bias and limited the global generalizability of findings. Additionally, grey literature was not included, and the divergence of identity constructs and measures prevented meta-analysis. Moreover, at the full-text screening stage, missing participant age ranges may have led to the exclusion of potentially relevant papers. Relatedly, while the review aimed to focus on the 16–24 age group, many papers included participants outside of this range. As a result, some of the measures assessed may not have been developmentally appropriate for AYA or aligned with the specific identity tasks characteristic of this life stage.

### Implications for future research, clinical practice, and measure development

Future research and measure development should be guided by the specific needs of both researchers and clinicians. To enhance the applicability and utility of existing measures, further psychometric validation is required, with appropriate modifications to ensure their relevance to clinical populations. At present, weaknesses in validity limit the extent to which identity measures can support accurate clinical assessment or detect meaningful change following intervention. Particular attention should be paid to the 16–24 age group, as this developmental stage presents unique identity-related challenges (Sawyer et al., 2018) that may not be adequately captured by tools developed for older populations. There is also a clear need for the development of new measures, or the redevelopment of existing measures, grounded in the lived experiences of AYA and clinical perspectives. This approach will help ensure content validity. Additionally, measures that are co-produced with individuals with lived experience and healthcare professionals are more likely to be clinically meaningful and better suited to inform interventions (Flake et al., 2017; Terwee et al., 2007). The development of such measures should follow established best practice guidelines (Mokkink et al., 2018; Vet et al., 2011).

Review findings demonstrated that while identity has been addressed in a range of papers involving individuals with LTC-P, notable gaps remain in the literature. Certain populations (such as chronic pain, e.g., fibromyalgia, endometriosis, and Ehlers–Danlos syndrome) were absent, suggesting a need for further research that includes these overlooked groups. Additionally, there is a particular need for the development of measures tailored to these under-represented LTC-P, which often span multiple diagnostic categories and are marked by diverse symptom presentations.

In addition, future papers should aim to evaluate key measurement properties that remain underexplored, including cross-cultural validity, measurement invariance, test–retest reliability, measurement error, criterion validity, and responsiveness. Without robust evidence for these properties, identity scores cannot be interpreted with confidence in research or clinical practice, nor used reliably to evaluate whether interventions are producing meaningful change. Notably, none of the reviewed measures examined the *content* of identity itself, an important and underdeveloped area that could serve as a valuable direction for future research (Galliher et al., 2017).

Finally, given the established relevance of identity to a range of health-related outcomes, including psychological well-being, treatment adherence, and quality of life among AYA with LTC-P, it is crucial that psychometrically robust, gold-standard measures are developed for specific clinical populations. Such measures are needed to accurately assess identity-related experiences in these contexts and to better inform the development of targeted interventions. The creation of these instruments should adhere to best practice guidelines for measure development set out by the COSMIN initiative (Mokkink et al., 2018; Vet et al., 2011) to ensure the psychometric and methodological rigor required of measures, particularly if identity is to be considered a clinical outcome.

## Conclusions

While the tentatively recommended IIQ and DIDS may be appropriate when their conceptualizations align with research aims, remaining measures within this review should be used with caution. Researchers must assess the relevance and comprehensiveness of any measure before applying it to a given population and ensure that its content is comprehensible to the target group, taking into account condition-specific factors.

## Supplementary Material

jsag001_Supplementary_Data

## Data Availability

Data available on request.
