# Supplementary material for: Measures of identity in adolescents/young adults with long-term physical health conditions: a systematic review
Source: J Pediatr Psychol. 2026 Feb 13;51(5):412–37. doi: 10.1093/jpepsy/jsag001 (PMC13221185; doi:10.1093/jpepsy/jsag001)
Supplement: jsag001_Supplementary_Data [file jsag001_supplementary_data.zip › jsag001_Supplementary_Data/jpepsy-2025-0199-File009.docx]

**General search terms:**

**Identity:** Identity OR “Identity Formation” OR “Identity Development” OR “Identity Exploration” OR “Illness Identity”

**Long Term Conditions – Physical:** "Long Term Condition" OR "Chronic condition" OR LTC OR “Recurrent Condition” OR “Chronic Disease” OR "Chronic Pain" OR "Persistent Pain" OR Diabetes OR Crohn's OR IBD or "Ulcerative Colitis" OR "Inflammatory Bowel Disease" OR CRPS OR "Complex Regional Pain Syndrome" OR Fibromyalgia OR Arthritis OR Endometriosis OR Migraine OR “Chronic daily headache” OR Osteoarthritis OR "Spina Bifida" OR Asthma OR Allergy OR “Atrial fibrillation” OR “Chronic Constipation” OR “Chronic Fatigue Syndrome” OR CFS OR ME OR “Myalgic encephalomyelitis” OR “Chronic Kidney Disease” OR “Congestive Heart Failure” OR Epilepsy OR Hypertension OR HIV OR “Irritable Bowel Disease” OR “Multiple Sclerosis” OR Anaemia OR Angiooedema OR “Sickle Cell” OR “Autoimmune Disorder*” OR “Autoimmune Disease” OR Lupus OR “Sjögrens syndrome” OR “Bronchopulmonary dysplasia” OR Cancer OR “Cardiac arrhythmias” OR “Coeliac Disease” OR “Coronary heart disease” OR “Cystic Fibrosis” OR “Endocrine disorder*” OR “Widespread pain” OR Gout OR “Heart Failure” OR “Lung Fibrosis” OR “Ehlers Danlos syndrome” OR haemophilia OR Rheumatoid OR Neurofibromatosis OR “Charcot-Marie-Tooth disease” OR Scoliosis OR scleroderma OR vasculitis OR “juvenile idiopathic arthritis” OR “rheumatoid arthritis” OR “congenital heart disease” OR thalassemia OR “chronic liver disease” OR cancer OR dermatitis OR eczema OR psoriasis

**Measure:** Surveys OR Questionnaires OR Psychometrics OR measure* OR tool* OR assessment OR scale* OR inventory OR index

**APA PsychNet:**

**Title**: Identity OR “Identity Formation” OR “Identity Development” OR “Identity Exploration” OR “Illness Identity” *AND* **Title**: "Long-term condition" OR "Long Term Condition" OR "Chronic condition" OR LTC OR “Recurrent Condition” OR “Chronic Disease” OR "Chronic Pain" OR "Persistent Pain" OR Diabetes OR Crohn's OR IBD or "Ulcerative Colitis" OR "Inflammatory Bowel Disease" OR CRPS OR "Complex Regional Pain Syndrome" OR Fibromyalgia OR Arthritis OR Endometriosis OR Migraine OR “Chronic daily headache” OR Osteoarthritis OR "Spina Bifida" OR Asthma OR Allergy OR “Atrial fibrillation” OR “Chronic Constipation” OR “Chronic Fatigue Syndrome” OR CFS OR ME OR “Myalgic encephalomyelitis” OR “Chronic Kidney Disease” OR “Congestive Heart Failure” OR Epilepsy OR Hypertension OR HIV OR “Irritable Bowel Disease” OR “Multiple Sclerosis” OR Anaemia OR Angiooedema OR “Sickle Cell” OR “Autoimmune Disorder*” OR “Autoimmune Disease” OR Lupus OR “Sjögrens syndrome” OR “Bronchopulmonary dysplasia” OR Cancer OR “Cardiac arrhythmias” OR “Coeliac Disease” OR “Coronary heart disease” OR “Cystic Fibrosis” OR “Endocrine disorder*” OR “Widespread pain” OR Gout OR “Heart Failure” OR “Lung Fibrosis” OR “Ehlers Danlos syndrome” OR haemophilia OR Rheumatoid OR Neurofibromatosis OR “Charcot-Marie-Tooth disease” OR Scoliosis OR scleroderma OR vasculitis OR “juvenile idiopathic arthritis” OR “rheumatoid arthritis” OR “congenital heart disease” OR thalassemia OR “chronic liver disease” OR cancer OR dermatitis OR eczema OR psoriasis *AND* **Title**: Surveys *OR* **Title**: Questionnaires *OR* **Title**: Psychometrics *OR* **Title**: measure* *OR* **Title**: tool* *OR* **Title**: assessment *OR* **Title**: scale* *OR* **Title**: inventory *OR* **Title**: index *OR* **Abstract**: Identity OR “Identity Formation” OR “Identity Development” OR “Identity Exploration” OR “Illness Identity” *AND* **Abstract**: : "Long-term condition" OR "Long Term Condition" OR "Chronic condition" OR LTC OR “Recurrent Condition” OR “Chronic Disease” OR "Chronic Pain" OR "Persistent Pain" OR Diabetes OR Crohn's OR IBD or "Ulcerative Colitis" OR "Inflammatory Bowel Disease" OR CRPS OR "Complex Regional Pain Syndrome" OR Fibromyalgia OR Arthritis OR Endometriosis OR Migraine OR “Chronic daily headache” OR Osteoarthritis OR "Spina Bifida" OR Asthma OR Allergy OR “Atrial fibrillation” OR “Chronic Constipation” OR “Chronic Fatigue Syndrome” OR CFS OR ME OR “Myalgic encephalomyelitis” OR “Chronic Kidney Disease” OR “Congestive Heart Failure” OR Epilepsy OR Hypertension OR HIV OR “Irritable Bowel Disease” OR “Multiple Sclerosis” OR Anaemia OR Angiooedema OR “Sickle Cell” OR “Autoimmune Disorder*” OR “Autoimmune Disease” OR Lupus OR “Sjögrens syndrome” OR “Bronchopulmonary dysplasia” OR Cancer OR “Cardiac arrhythmias” OR “Coeliac Disease” OR “Coronary heart disease” OR “Cystic Fibrosis” OR “Endocrine disorder*” OR “Widespread pain” OR Gout OR “Heart Failure” OR “Lung Fibrosis” OR “Ehlers Danlos syndrome” OR haemophilia OR Rheumatoid OR Neurofibromatosis OR “Charcot-Marie-Tooth disease” OR Scoliosis OR scleroderma OR vasculitis OR “juvenile idiopathic arthritis” OR “rheumatoid arthritis” OR “congenital heart disease” OR thalassemia OR “chronic liver disease” OR cancer OR dermatitis OR eczema OR psoriasis *AND* **Abstract**: Surveys *OR* **Abstract**: Questionnaires *OR* **Abstract**: Psychometrics *OR* **Abstract**: measure* *OR* **Abstract**: tool* *OR* **Abstract**: assessment *OR* **Abstract**: scale* *OR* **Abstract**: inventory *OR* **Abstract**: index *AND* **Peer-Reviewed Journals only**

**PubMed:**

((Identity[Title/Abstract] OR "Identity Formation"[Title/Abstract] OR "Identity Development"[Title/Abstract] OR "Identity Exploration"[Title/Abstract] OR "Illness Identity"[Title/Abstract]) AND ("Long Term Condition"[Title/Abstract] OR "Chronic condition"[Title/Abstract] OR LTC[Title/Abstract] OR "Recurrent Condition"[Title/Abstract] OR "Chronic Disease"[Title/Abstract] OR "Chronic Pain"[Title/Abstract] OR "Persistent Pain"[Title/Abstract] OR Diabetes[Title/Abstract] OR Crohn's[Title/Abstract] OR IBD[Title/Abstract] OR "Ulcerative Colitis"[Title/Abstract] OR "Inflammatory Bowel Disease"[Title/Abstract] OR CRPS[Title/Abstract] OR "Complex Regional Pain Syndrome"[Title/Abstract] OR Fibromyalgia[Title/Abstract] OR Arthritis[Title/Abstract] OR Endometriosis[Title/Abstract] OR Migraine[Title/Abstract] OR "Chronic daily headache"[Title/Abstract] OR Osteoarthritis[Title/Abstract] OR "Spina Bifida"[Title/Abstract] OR Asthma[Title/Abstract] OR Allergy[Title/Abstract] OR "Atrial fibrillation"[Title/Abstract] OR "Chronic Constipation"[Title/Abstract] OR "Chronic Fatigue Syndrome"[Title/Abstract] OR CFS[Title/Abstract] OR ME[Title/Abstract] OR "Myalgic encephalomyelitis"[Title/Abstract] OR "Chronic Kidney Disease"[Title/Abstract] OR "Congestive Heart Failure"[Title/Abstract] OR Epilepsy[Title/Abstract] OR Hypertension[Title/Abstract] OR HIV[Title/Abstract] OR "Irritable Bowel Disease"[Title/Abstract] OR "Multiple Sclerosis"[Title/Abstract] OR Anaemia[Title/Abstract] OR Angiooedema[Title/Abstract] OR "Sickle Cell"[Title/Abstract] OR "Autoimmune Disorder*"[Title/Abstract] OR "Autoimmune Disease"[Title/Abstract] OR Lupus[Title/Abstract] OR "Sjögrens syndrome"[Title/Abstract] OR "Bronchopulmonary dysplasia"[Title/Abstract] OR Cancer[Title/Abstract] OR "Cardiac arrhythmias"[Title/Abstract] OR "Coeliac Disease"[Title/Abstract] OR "Coronary heart disease"[Title/Abstract] OR "Cystic Fibrosis"[Title/Abstract] OR "Endocrine disorder*"[Title/Abstract] OR "Widespread pain"[Title/Abstract] OR Gout[Title/Abstract] OR "Heart Failure"[Title/Abstract] OR "Lung Fibrosis"[Title/Abstract] OR "Ehlers Danlos syndrome"[Title/Abstract] OR haemophilia[Title/Abstract] OR Rheumatoid[Title/Abstract] OR Neurofibromatosis[Title/Abstract] OR "Charcot-Marie-Tooth disease"[Title/Abstract] OR Scoliosis[Title/Abstract] OR scleroderma[Title/Abstract] OR vasculitis[Title/Abstract] OR "juvenile idiopathic arthritis"[Title/Abstract] OR "rheumatoid arthritis"[Title/Abstract] OR "congenital heart disease"[Title/Abstract] OR thalassemia[Title/Abstract] OR "chronic liver disease"[Title/Abstract] OR cancer[Title/Abstract] OR dermatitis[Title/Abstract] OR eczema[Title/Abstract] OR psoriasis[Title/Abstract])) AND (Surveys[Title/Abstract] OR Questionnaires[Title/Abstract] OR Psychometrics[Title/Abstract] OR measure*[Title/Abstract] OR tool*[Title/Abstract] OR assessment[Title/Abstract] OR scale*[Title/Abstract] OR inventory[Title/Abstract] OR index[Title/Abstract])

**Web of Science:**

(((((TI=(Identity OR “Identity Formation” OR “Identity Development” OR “Identity Exploration” OR “Illness Identity”)) AND TI=("Long-term condition" OR "Long Term Condition" OR "Chronic condition" OR LTC OR “Recurrent Condition” OR “Chronic Disease” OR "Chronic Pain" OR "Persistent Pain" OR Diabetes OR Crohn's OR IBD or "Ulcerative Colitis" OR "Inflammatory Bowel Disease" OR CRPS OR "Complex Regional Pain Syndrome" OR Fibromyalgia OR Arthritis OR Endometriosis OR Migraine OR “Chronic daily headache” OR Osteoarthritis OR "Spina Bifida" OR Asthma OR Allergy OR “Atrial fibrillation” OR “Chronic Constipation” OR “Chronic Fatigue Syndrome” OR CFS OR ME OR “Myalgic encephalomyelitis” OR “Chronic Kidney Disease” OR “Congestive Heart Failure” OR Epilepsy OR Hypertension OR HIV OR “Irritable Bowel Disease” OR “Multiple Sclerosis” OR Anaemia OR Angiooedema OR “Sickle Cell” OR “Autoimmune Disorder*” OR “Autoimmune Disease” OR Lupus OR “Sjögrens syndrome” OR “Bronchopulmonary dysplasia” OR Cancer OR “Cardiac arrhythmias” OR “Coeliac Disease” OR “Coronary heart disease” OR “Cystic Fibrosis” OR “Endocrine disorder*” OR “Widespread pain” OR Gout OR “Heart Failure” OR “Lung Fibrosis” OR “Ehlers Danlos syndrome” OR haemophilia OR Rheumatoid OR Neurofibromatosis OR “Charcot-Marie-Tooth disease” OR Scoliosis OR scleroderma OR vasculitis OR “juvenile idiopathic arthritis” OR “rheumatoid arthritis” OR “congenital heart disease” OR thalassemia OR “chronic liver disease” OR cancer OR dermatitis OR eczema OR psoriasis)) AND TI=(Surveys OR Questionnaires OR Psychometrics OR measure* OR tool* OR assessment OR scale* OR inventory OR index)) OR AB=(Identity OR “Identity Formation” OR “Identity Development” OR “Identity Exploration” OR “Illness Identity” )) AND AB=("Long-term condition" OR "Long Term Condition" OR "Chronic condition" OR LTC OR “Recurrent Condition” OR “Chronic Disease” OR "Chronic Pain" OR "Persistent Pain" OR Diabetes OR Crohn's OR IBD or "Ulcerative Colitis" OR "Inflammatory Bowel Disease" OR CRPS OR "Complex Regional Pain Syndrome" OR Fibromyalgia OR Arthritis OR Endometriosis OR Migraine OR “Chronic daily headache” OR Osteoarthritis OR "Spina Bifida" OR Asthma OR Allergy OR “Atrial fibrillation” OR “Chronic Constipation” OR “Chronic Fatigue Syndrome” OR CFS OR ME OR “Myalgic encephalomyelitis” OR “Chronic Kidney Disease” OR “Congestive Heart Failure” OR Epilepsy OR Hypertension OR HIV OR “Irritable Bowel Disease” OR “Multiple Sclerosis” OR Anaemia OR Angiooedema OR “Sickle Cell” OR “Autoimmune Disorder*” OR “Autoimmune Disease” OR Lupus OR “Sjögrens syndrome” OR “Bronchopulmonary dysplasia” OR Cancer OR “Cardiac arrhythmias” OR “Coeliac Disease” OR “Coronary heart disease” OR “Cystic Fibrosis” OR “Endocrine disorder*” OR “Widespread pain” OR Gout OR “Heart Failure” OR “Lung Fibrosis” OR “Ehlers Danlos syndrome” OR haemophilia OR Rheumatoid OR Neurofibromatosis OR “Charcot-Marie-Tooth disease” OR Scoliosis OR scleroderma OR vasculitis OR “juvenile idiopathic arthritis” OR “rheumatoid arthritis” OR “congenital heart disease” OR thalassemia OR “chronic liver disease” OR cancer OR dermatitis OR eczema OR psoriasis)) AND AB=(Surveys OR Questionnaires OR Psychometrics OR measure* OR tool* OR assessment OR scale* OR inventory OR index)

**SCOPUS:**

((TITLE(Identity OR "Identity Formation" OR "Identity Development" OR "Identity Exploration" OR "Illness Identity")) AND (TITLE("Long Term Condition" OR "Chronic condition" OR LTC OR "Recurrent Condition" OR "Chronic Disease" OR "Chronic Pain" OR "Persistent Pain" OR Diabetes OR Crohn's OR IBD or "Ulcerative Colitis" OR "Inflammatory Bowel Disease" OR CRPS OR "Complex Regional Pain Syndrome" OR Fibromyalgia OR Arthritis OR Endometriosis OR Migraine OR "Chronic daily headache" OR Osteoarthritis OR "Spina Bifida" OR Asthma OR Allergy OR "Atrial fibrillation" OR "Chronic Constipation" OR "Chronic Fatigue Syndrome" OR CFS OR ME OR "Myalgic encephalomyelitis" OR "Chronic Kidney Disease" OR "Congestive Heart Failure" OR Epilepsy OR Hypertension OR HIV OR "Irritable Bowel Disease" OR "Multiple Sclerosis" OR Anaemia OR Angiooedema OR "Sickle Cell" OR "Autoimmune Disorder*" OR "Autoimmune Disease" OR Lupus OR "Sjögrens syndrome" OR "Bronchopulmonary dysplasia" OR Cancer OR "Cardiac arrhythmias" OR "Coeliac Disease" OR "Coronary heart disease" OR "Cystic Fibrosis" OR "Endocrine disorder*" OR "Widespread pain" OR Gout OR "Heart Failure" OR "Lung Fibrosis" OR "Ehlers Danlos syndrome" OR haemophilia OR Rheumatoid OR Neurofibromatosis OR "Charcot-Marie-Tooth disease" OR Scoliosis OR scleroderma OR vasculitis OR "juvenile idiopathic arthritis" OR "rheumatoid arthritis" OR "congenital heart disease" OR thalassemia OR "chronic liver disease" OR cancer OR dermatitis OR eczema OR psoriasis)) AND (TITLE(Surveys OR Questionnaires OR Psychometrics OR measure* OR tool* OR assessment OR scale* OR inventory OR index))) OR ((ABS(Identity OR "Identity Formation" OR "Identity Development" OR "Identity Exploration" OR "Illness Identity")) AND (ABS("Long Term Condition" OR "Chronic condition" OR LTC OR "Recurrent Condition" OR "Chronic Disease" OR "Chronic Pain" OR "Persistent Pain" OR Diabetes OR Crohn's OR IBD or "Ulcerative Colitis" OR "Inflammatory Bowel Disease" OR CRPS OR "Complex Regional Pain Syndrome" OR Fibromyalgia OR Arthritis OR Endometriosis OR Migraine OR "Chronic daily headache" OR Osteoarthritis OR "Spina Bifida" OR Asthma OR Allergy OR "Atrial fibrillation" OR "Chronic Constipation" OR "Chronic Fatigue Syndrome" OR CFS OR ME OR "Myalgic encephalomyelitis" OR "Chronic Kidney Disease" OR "Congestive Heart Failure" OR Epilepsy OR Hypertension OR HIV OR "Irritable Bowel Disease" OR "Multiple Sclerosis" OR Anaemia OR Angiooedema OR "Sickle Cell" OR "Autoimmune Disorder*" OR "Autoimmune Disease" OR Lupus OR "Sjögrens syndrome" OR "Bronchopulmonary dysplasia" OR Cancer OR "Cardiac arrhythmias" OR "Coeliac Disease" OR "Coronary heart disease" OR "Cystic Fibrosis" OR "Endocrine disorder*" OR "Widespread pain" OR Gout OR "Heart Failure" OR "Lung Fibrosis" OR "Ehlers Danlos syndrome" OR haemophilia OR Rheumatoid OR Neurofibromatosis OR "Charcot-Marie-Tooth disease" OR Scoliosis OR scleroderma OR vasculitis OR "juvenile idiopathic arthritis" OR "rheumatoid arthritis" OR "congenital heart disease" OR thalassemia OR "chronic liver disease" OR cancer OR dermatitis OR eczema OR psoriasis)) AND (ABS(Surveys OR Questionnaires OR Psychometrics OR measure* OR tool* OR assessment OR scale* OR inventory OR index))) AND ( LIMIT-TO ( DOCTYPE,"ar" ) )

**CINAHL:**

( TI(Identity OR "Identity Formation" OR "Identity Development" OR "Identity Exploration" OR "Illness Identity") OR AB(Identity OR "Identity Formation" OR "Identity Development" OR "Identity Exploration" OR "Illness Identity") ) AND ( TI("Long Term Condition" OR "Chronic condition" OR LTC OR "Recurrent Condition" OR "Chronic Disease" OR "Chronic Pain" OR "Persistent Pain" OR Diabetes OR Crohn's OR IBD OR "Ulcerative Colitis" OR "Inflammatory Bowel Disease" OR CRPS OR "Complex Regional Pain Syndrome" OR Fibromyalgia OR Arthritis OR Endometriosis OR Migraine OR "Chronic daily headache" OR Osteoarthritis OR "Spina Bifida" OR Asthma OR Allergy OR "Atrial fibrillation" OR "Chronic Constipation" OR "Chronic Fatigue Syndrome" OR CFS OR ME OR "Myalgic encephalomyelitis" OR "Chronic Kidney Disease" OR "Congestive Heart Failure" OR Epilepsy OR Hypertension OR HIV OR "Irritable Bowel Disease" OR "Multiple Sclerosis" OR Anaemia OR Angiooedema OR "Sickle Cell" OR "Autoimmune Disorder*" OR "Autoimmune Disease" OR Lupus OR "Sjögrens syndrome" OR "Bronchopulmonary dysplasia" OR Cancer OR "Cardiac arrhythmias" OR "Coeliac Disease" OR "Coronary heart disease" OR "Cystic Fibrosis" OR "Endocrine disorder*" OR "Widespread pain" OR Gout OR "Heart Failure" OR "Lung Fibrosis" OR "Ehlers Danlos syndrome" OR haemophilia OR Rheumatoid OR Neurofibromatosis OR "Charcot-Marie-Tooth disease" OR Scoliosis OR scleroderma OR vasculitis OR "juvenile idiopathic arthritis" OR "rheumatoid arthritis" OR "congenital heart disease" OR thalassemia OR "chronic liver disease" OR dermatitis OR eczema OR psoriasis) OR AB("Long Term Condition" OR "Chronic condition" OR LTC OR "Recurrent Condition" OR "Chronic Disease" OR "Chronic Pain" OR "Persistent Pain" OR Diabetes OR Crohn's OR IBD OR "Ulcerative Colitis" OR "Inflammatory Bowel Disease" OR CRPS OR "Complex Regional Pain Syndrome" OR Fibromyalgia OR Arthritis OR Endometriosis OR Migraine OR "Chronic daily headache" OR Osteoarthritis OR "Spina Bifida" OR Asthma OR Allergy OR "Atrial fibrillation" OR "Chronic Constipation" OR "Chronic Fatigue Syndrome" OR CFS OR ME OR "Myalgic encephalomyelitis" OR "Chronic Kidney Disease" OR "Congestive Heart Failure" OR Epilepsy OR Hypertension OR HIV OR "Irritable Bowel Disease" OR "Multiple Sclerosis" OR Anaemia OR Angiooedema OR "Sickle Cell" OR "Autoimmune Disorder*" OR "Autoimmune Disease" OR Lupus OR "Sjögrens syndrome" OR "Bronchopulmonary dysplasia" OR Cancer OR "Cardiac arrhythmias" OR "Coeliac Disease" OR "Coronary heart disease" OR "Cystic Fibrosis" OR "Endocrine disorder*" OR "Widespread pain" OR Gout OR "Heart Failure" OR "Lung Fibrosis" OR "Ehlers Danlos syndrome" OR haemophilia OR Rheumatoid OR Neurofibromatosis OR "Charcot-Marie-Tooth disease" OR Scoliosis OR scleroderma OR vasculitis OR "juvenile idiopathic arthritis" OR "rheumatoid arthritis" OR "congenital heart disease" OR thalassemia OR "chronic liver disease" OR dermatitis OR eczema OR psoriasis) ) AND ( TI(measure* OR tool* OR assessment OR scale* OR inventory OR index) OR AB(measure* OR tool* OR assessment OR scale* OR inventory OR index) )
