# Supplementary material for: Measures of identity in adolescents/young adults with long-term physical health conditions: a systematic review
Source: J Pediatr Psychol. 2026 Feb 13;51(5):412–37. doi: 10.1093/jpepsy/jsag001 (PMC13221185; doi:10.1093/jpepsy/jsag001)
Supplement: jsag001_Supplementary_Data [file jsag001_supplementary_data.zip › jsag001_Supplementary_Data/jpepsy-2025-0199-File010.docx]

**Supplementary Materials 2:** Criteria for good measurement properties (Mokkink et al., 2017)

| **Measurement property** | **Rating^1^** | **Criteria** |
| --- | --- | --- |
| Structural validity | **+** | **CTT:**  **CFA:** CFI or TLI or comparable measure >0.95 OR RMSEA <0.06 OR SRMR <0.08^2^  **IRT/Rasch:**  No violation of unidimensionality^3^: CFI or TLI or comparable measure >0.95 OR RMSEA <0.06 OR SRMR <0.08  *AND*  no violation of local independence: residual correlations among the items after controlling for the dominant factor <0.20 OR Q3's <0.37  *AND*  no violation of monotonicity: adequate looking graphs OR item scalability >0.30  *AND*  adequate model fit:  IRT: χ2 >0.01  Rasch: infit and outfit mean squares ≥ 0.5 and ≤ 1.5 OR Z‐standardized values > ‐2 and <2 |
|  | ? | CTT: Not all information for ‘+’ reported  IRT/Rasch: Model fit not reported |
|  | **-** | Criteria for ‘+’ not met |
| Internal consistency | + | At least low evidence4 for sufficient structural validity^5^ AND Cronbach's alpha(s) ≥ 0.70 for each unidimensional scale or subscale^6^ |
|  | ? | Criteria for “At least low evidence^4^ for sufficient structural validity^5^” not met |
|  | - | At least low evidence^4^ for sufficient structural validity^5^ AND Cronbach’s alpha(s) < 0.70 for each unidimensional scale or subscale^6^ |
| Reliability | + | ICC or weighted Kappa ≥ 0.70 |
|  | ? | ICC or weighted Kappa not reported |
|  | - | ICC or weighted Kappa < 0.70 |
| Measurement error | + | SDC or LoA < MIC^5^ |
|  | ? | MIC not defined |
|  | - | SDC or LoA > MIC^5^ |
| Hypotheses testing for  construct validity | + | The result is in accordance with the hypothesis^7^ |
|  | ? | No hypothesis defined (by the review team) |
|  | - | The result is not in accordance with the hypothesis^7^ |
| Cross‐cultural  validity\measurement  invariance | + | No important differences found between group factors (such as age, gender, language) in multiple group factor analysis OR no important DIF for group factors (McFadden's R^2^ < 0.02) |
|  | ? | No multiple group factor analysis OR DIF analysis performed |
|  | - | Important differences between group factors OR DIF was found |
| Criterion validity | + | Correlation with gold standard ≥ 0.70 OR AUC ≥ 0.70 |
|  | ? | Not all information for ‘+’ reported |
|  | - | Correlation with gold standard < 0.70 OR AUC < 0.70 |
| Responsiveness | + | The result is in accordance with the hypothesis^7^ OR AUC ≥ 0.70 |
|  | ? | No hypothesis defined (by the review team) |
|  | - | The result is not in accordance with the hypothesis^7^ OR AUC <0.70 |

AUC = area under the curve, CFA = confirmatory factor analysis, CFI = comparative fit index, CTT= classical test theory, DIF = differential item functioning, ICC = intraclass correlation coefficient, IRT = item response theory, LoA = limits of agreement, MIC = minimal important change, RMSEA: Root Mean Square Error of Approximation, SEM = Standard Error of Measurement, SDC= smallest detectable change, SRMR: Standardized Root Mean Residuals, TLI = Tucker‐Lewis index

^1^ “+” = sufficient, ” –“ = insufficient, “?” = indeterminate

2 To rate the quality of the summary score, the factor structures should be equal across studies

^3^ unidimensionality refers to a factor analysis per subscale, while structural validity refers to a factor analysis of a (multidimensional) patient‐reported outcome measure

^4^ As defined by grading the evidence according to the GRADE approach

^5^ This evidence may come from different studies

^6^ The criteria ‘Cronbach alpha < 0.95’ was deleted, as this is relevant in the development phase of a PROM and not when evaluating an existing PROM.

^7^ The results of all studies should be taken together and it should then be decided if 75% of the results are in accordance with the hypotheses
